# Bright Self‐Trapped Exciton Emission of CsPbBr_3_@CsPb_2_Br_5_ Nanostructures Created by Dissolution and Recrystallization of Hydrophilic Cu:CsPbBr_3_

**DOI:** 10.1002/smll.73956

**Published:** 2026-05-26

**Authors:** Wenbin Shi, Xiao Zhang, Hsueh Shih Chen, San Ping Jiang, Ping Yang

**Affiliations:** ^1^ School of Material Science and Engineering University of Jinan Jinan P. R. China; ^2^ Advanced Institute For Materials Research (WPI‐AIMR) Tohoku University Sendai Japan; ^3^ Department of Materials Science and Engineering National Tsing Hua University Hsinchu Taiwan; ^4^ WA School of Mines: Minerals, Energy and Chemical Engineering Curtin University Perth Australia

**Keywords:** CsPbBr_3_, CsPb_2_Br_5_, nanoarchitectonics, photoluminescence, self‐trapped exciton

## Abstract

Nanoarchitectonics provide significant opportunities to endow materials with special photoluminescence (PL) properties. In this paper, novel self‐trapped exciton (STE) orange‐red emission is observed by creating CsPbBr_3_@CsPb_2_Br_5_ nanostructures through controlling the dissolution and recrystallization of Cu:CsPbBr_3_. Due to the dissolution and phase transfer of CsPbBr_3_, CsPbBr_3_@CsPb_2_Br_5_ nanostructures are formed. Cu^+^ instead of Pb^2+^ induced significant lattice distortion and formed a multi‐core@shell configuration, promoting STE emission center establishment and enabling the broad orange‐red PL peak (620 nm) of CsPbBr_3_ components with a PL quantum yield (PLQY) of 45.6%. The CsPb_2_Br_5_ shell effectively isolated the nanostructure from moisture and oxygen, resulting in extraordinary stability under ambient, ultraviolet light irradiation, and aqueous solutions. Cu^+^ component is a key for the STE emission because Na:CsPbBr_3_@CsPb_2_Br_5_ sample prepared using the same procedure revealed a narrow green PL peak (516 nm) even though the PLQY increased to 82.8% from 55.4%. Na:CsPbBr_3_@CsPb_2_Br_5_ sample exhibited a single‐core@shell structure, in which Na^+^ tends to occupy interstitial sites, effectively passivating deep‐level defects. Because of high stability, an light emitting diode was fabricated using Cu:CsPbBr_3_@CsPb_2_Br_5_ to show stable warm‐orange emission with a correlated color temperature of 1430 K, and a color rendering index of 62, suggesting potential solid‐state lighting applications.

## Graphical abstract

This work reports a differentiated ion‐doping strategy to engineer the self‐trapped exciton emission of CsPbBr_3_@CsPb_2_Br_5_ nanostructures, in which bright orange emission is obtained by the dissolution and recrystallization of Cu:CsPbBr_3_ in water. Lattice‐incorporated Cu components create self‐trapped exciton centers because of bright green emission observed in the case of Na^+^ instead of Cu^+^ ions. The protective CsPb_2_Br_5_ shell ensured excellent environmental stability. A fabricated orange‐red LED demonstrated the potential application of doped CsPbBr_3_@CsPb_2_Br_5_ nanostructures in stable solid‐state lighting.

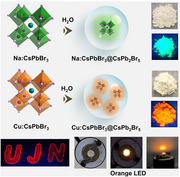

## 1 Introduction

As an important members of the metal halide perovskite family, CsPbBr_3_ nanocrystals (NCs) have attracted extensive attention over the past decade owing to their outstanding optoelectronic performance [1, 2, 3]. These NCs synthesized in hydrophobic solvents revealed blue to green photoluminescence (PL), narrow PL spectra, and high PL quantum yields (PLQY) [4, 5, 6]. However, these hydrophobic NCs revealed poor stability upon exposure to humidity, light, or heat environmental [7, 8, 9]. The ionic crystal nature of CsPbBr_3_ renders it highly susceptible to ion migration, ligand desorption, crystal degradation, and even phase transitions under light irradiation, thermal stress, ambient moisture/oxygen, and electric fields [10, 11, 12]. Such instability caused rapid quenching of luminescence and severely limit their applications. The growth of stable shell on these NCs can significantly influence the migration of charge carriers in CsPbBr_3_NCs, improve PL performance, and increase stability. Furthermore, the construction of inherently robust CsPbBr_3_ based nanostructures becomes a hot topic to improve luminescence performance, study PL activities, and get significant applications.

Nanoarchitectonics as a significant concept supplied efficient approaches to address the stability and find novel PL activity for CsPbBr_3_ study [13, 14, 15, 16, 17]. For example, the construction of core‐shell nanostructures has emerged as an effective material design strategy [15]. Encapsulating CsPbBr_3_ NCs within a CsPb_2_Br_5_ shell, which possesses a wider bandgap and higher chemical stability, forming a CsPbBr_3_@CsPb_2_Br_5_ type‐I core‐shell heterostructure offers distinct advantages [18]. These nanostructures physically isolate the CsPbBr_3_ core from environmental degradation, owing to the type‐I band alignment, confine photogenerated carriers within the core. This effectively suppresses non‐radiative recombination, thereby simultaneously enhancing both the chemical stability and PL efficiency of the material. Simultaneously, lead halide perovskite systems exhibit another important luminescence pathway due to their “soft‐lattice” nature and strong electron‐phonon coupling, namely the generation of efficient broad band emission through self‐trapped excitons (STE) [19, 20, 21, 22]. STE form when photogenerated excitons induce transient local lattice distortion and become trapped in the resulting potential well. Their radiative recombination typically produces emission characterized by a large Stokes shift and a broad spectral range covering the entire visible region. This property enables single‐component lead halide perovskites to generate white‐light emission directly without the need for multi‐color compounding, providing a breakthrough material platform for developing structurally simple, next‐generation, single‐component white light emitting diode (LED) with controllable color temperature. In recent years, inducing efficient STE emission in lead halide perovskites through compositional engineering, dimensional modulation, and specific ion doping has become a forefront research focus in this field [23, 24].

Although CsPbBr_3_@CsPb_2_Br_5_ core‐shell nanostructures have demonstrated progress in terms of stability and defect passivation, the PL performance remains inherently constrained by the intrinsic band‐edge emission (≈510 nm) of CsPbBr_3_ cores. This poses inherent limitations for further improving the PLQY and expanding the range of PL peak wavelengths [25, 26]. To overcome these constraints, introducing ion doping engineering within the core‐shell nanostructure becomes a key strategy for achieving precise performance tuning and functionalization. Two distinct ion introduction strategies were employed to realize differentiated modulation of the luminescence properties of the core‐shell structure in this study. First, Na^+^ was introduced to prepare Na:CsPbBr_3_@CsPb_2_Br_5_. Based on analysis of ionic radius and charge matching, Na^+^ tends to occupy interstitial sites or accumulate at the core‐shell interface, rather than directly substituting lattice sites. This occupancy mode effectively fills halogen vacancies, inhibits ion migration, and thereby passivates deep‐level defects [27, 28]. Thus, non‐radiative recombination channels were significantly decreased to improve PL efficiency and maintained the characteristic green emission. In addition, the incorporation of Cu^+^ in CsPbBr_3_@CsPb_2_Br_5_ resulted in significant perturbation to the local electronic environment of the [PbBr_6_]^4−^ octahedral units, leading to dynamic Jahn‐Teller type lattice distortion. This persistent local structural distortion greatly enhances the strength of electron‐phonon coupling, creating the necessary conditions for exciton self‐trapping and thereby stably introducing an efficient STE luminescent state into the material [29]. Macroscopically, this is manifested by a red‐shift of the PL peak from the intrinsic band‐edge emission at 510 to 620 nm, accompanied by a transformation into a typical broad emission spectrum [30, 31]. This efficient broad emission band from a single material offers significant advantages for LED applications. It fundamentally avoids the issue of color drift caused by differential degradation rates of different components, providing an ideal material foundation for manufacturing LED with high color rendering index and high stability.

To address the challenge on insufficient stability and limited tunability in luminescent performance for CsPbBr_3_ NCs in LED applications, this paper constructs a robust CsPbBr_3_@CsPb_2_Br_5_ core‐shell platform to systematically investigate the distinct mechanisms through Na^+^ interstitial passivation and Cu^+^ lattice distortion differentially to affect the PL performance. CsPbBr_3_@CsPb_2_Br_5_ nanostructures were fabricated by the dissolution and phase transfer of CsPbBr_3_ via a two‐step aqueous synthesis. Na^+^ modification enhanced the intrinsic green PL via a defect‐passivation mechanism. In contrast, Cu^+^ doping successfully establishes STE emission centers within the core by inducing dynamic lattice distortion, leading to a mechanistic transition and spectral tuning from narrow‐band green to broad‐band orange‐red emission. These results supply an effective synergistic strategy combining core‐shell structuring with ion engineering for developing high‐performance and highly stable perovskite luminescent materials, and also deepens the understanding of the complex optoelectronic properties of lead halide perovskites by comparatively revealing the distinct influences of defect passivation and exciton self‐trapping mechanisms on emission behavior within a single system. These findings hold significant scientific implications for advancing their practical applications in next‐generation displays and solid‐state lighting.

## 2 Results and Discussion

**FIGURE 1:**
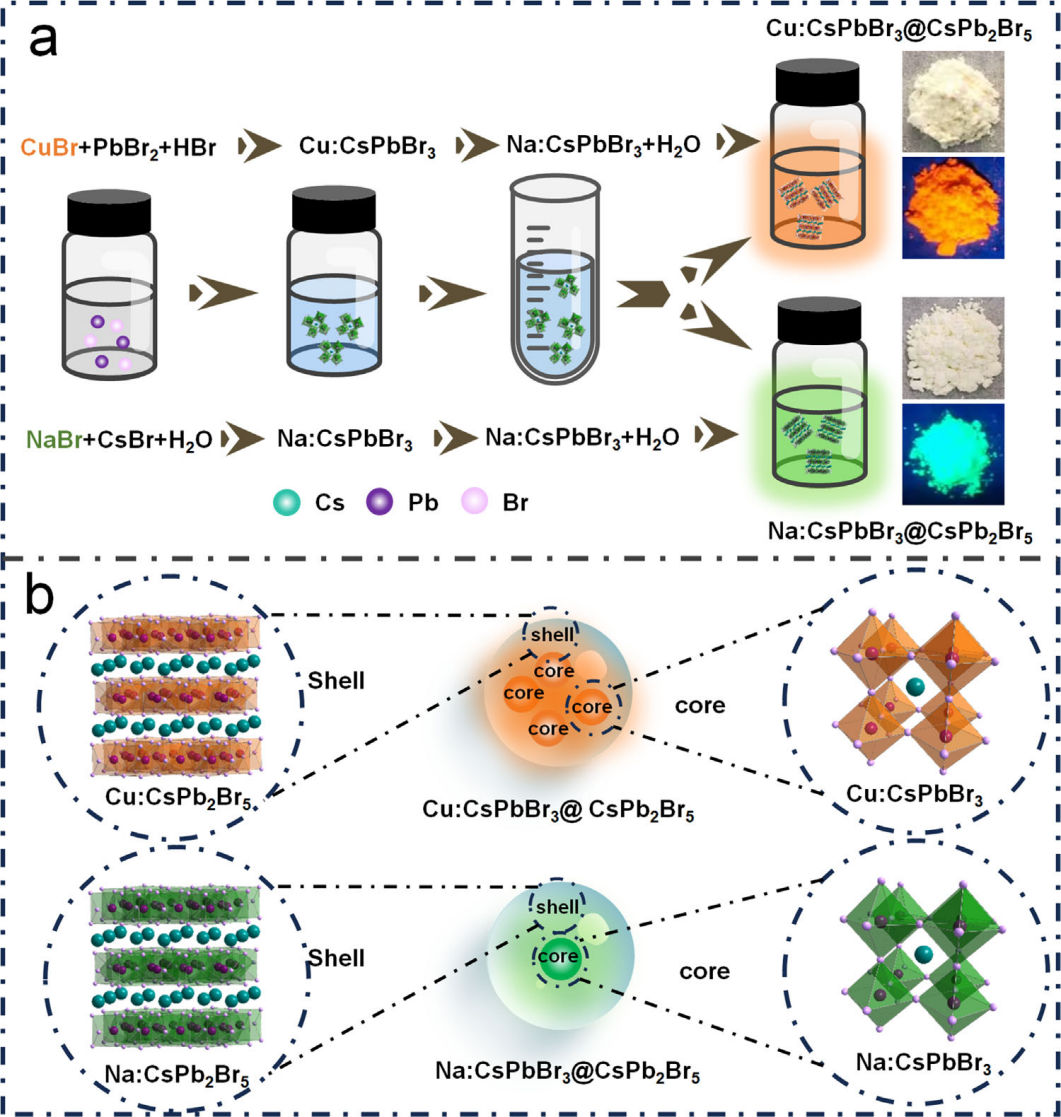
(a) Schematic illustration of preparation process of samples. (b) Microstructure of Na:CsPbBr_3_@CsPb_2_Br_5_ and Cu:CsPbBr_3_@CsPb_2_Br_5_.

Leveraging the significant solubility difference of precursors in specific solvents, CsPbBr_3_@CsPb_2_Br_5_ NCs with a core‐shell structure were synthesized via a two‐step route in hydrophilic solutions to realize the dissolution and the phase transfer of CsPbBr_3_. Figure 1a shows the synthesis procedure of CsPbBr_3_@CsPb_2_Br_5_ core‐shell nanostructures with distinct micro‐morphologies via Na and Cu doping strategies. PbBr_2_ was dissolved in hydrobromic acid reacted rapidly with CsBr in deionized water, yielding orange CsPbBr_3_ powders. Subsequent water treatment induced the rapid dissolution of surface CsBr and the phase transformation of CsPbBr_3_ into CsPb_2_Br_5_, ultimately forming CsPbBr_3_@CsPb_2_Br_5_ with a core‐shell architecture. Doping ions (Na^+^ or Cu^+^) were introduced into the precursor solutions, allowing them to incorporate into the lattice during nucleation and growth, ultimately yielding Na:CsPbBr_3_@CsPb_2_Br_5_ and Cu:CsPbBr_3_@CsPb_2_Br_5_ nanostructures. Detailed synthesis parameters of samples are provided in Table S1.

The incorporation sites of Na^+^ and Cu^+^ govern their behavior during crystallization, which in turn critically influences the nucleation mode of CsPbBr_3_ core and the growth pattern of CsPb_2_Br_5_ shell during phase transformation. These differences ultimately lead to two distinct heterostructures, “single‐core@shell” and “multi‐core@shell” as indicated in Figure 1b. Although the small ionic radius of Na^+^ makes direct substitution for Cs^+^ in the lattice difficult, its presence in the crystallization environment likely acts as a surface modifier or crystallization‐regulating agent. This contributes to the formation of well‐dispersed and size‐uniform CsPbBr_3_ NCs. Upon water treatment, partial dissolution of surface CsBr on these isolated NCs promotes their phase transformation, resulting in a continuous CsPb_2_Br_5_ shell that encapsulates individual CsPbBr_3_ cores. In contrast, for the Cu^+^ doped system, CuBr was introduced into the PbBr_2_ precursor. Cu^+^ exhibits a stronger tendency to coordinate with Br^−^, and its presence likely significantly alters the surface characteristics and aggregation propensity of CsPbBr_3_ NCs, inducing the formation of multi‑core aggregates during the precipitation stage. Throughout the aqueous phase treatment, these aggregates evolve into a composite nanostructure in which multiple CsPbBr_3_ cores are encapsulated by a shared CsPb_2_Br_5_ shell.

**FIGURE 2:**
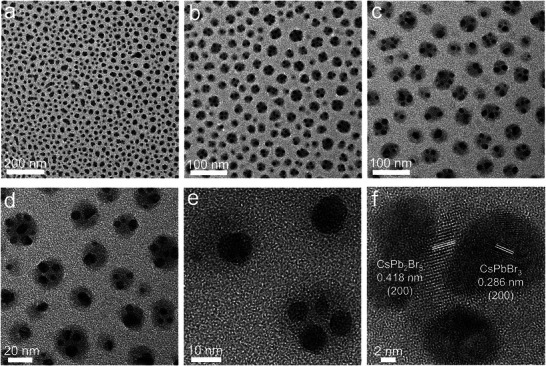
(a–f) TEM images of Cu:CsPbBr_3_@CsPb_2_Br_5_ at different magnifications. The lattice fringes of CsPbBr_3_ and CsPb_2_Br_5_ were clearly observed in (f).

Figure 2 shows the transmission electron microscopy (TEM) images of sample Cu:CsPbBr_3_@CsPb_2_Br_5_. Although uniformly spherical particles were observed in Figure 2a–d, the spherical particle included several cores. Multiple CsPbBr_3_ cores were encapsulated within each particle, forming a unique “multi‐core@shell” structure (Figure 2c,d). The High‐resolution TEM (HRTEM) images in Figure 2e,f show that specimen revealed fine crystallinity in both core and shell regions (Figure 2f). The lattice spacing in the core region is 0.286 nm, corresponding to the (200) plane of CsPbBr_3_. This value is smaller than that of the undoped sample, suggesting that the small Cu^+^ ions can substitute Pb^2+^ sites, inducing lattice distortion and compression. The shell region revealed a lattice spacing of 0.418 nm, assigned to the (200) plane of CsPb_2_Br_5_ which also exhibits a slight contraction, further supporting the structural modulation induced by Cu doping. Particle‐size statistics indicate an average diameter of 27.7 nm, with the CsPbBr_3_ cores averaging about 7.0 nm (Figure S1). The scanning electron microscopy (SEM) images and element mapping (Figure S2) confirm the homogeneous distribution of element Cs, Pb, Br, and Cu in the sample. The ubiquitous presence of Cu signals verifies the successful incorporation of Cu in CsPbBr_3_@CsPb_2_Br_5_ nanostructures.

**FIGURE 3:**
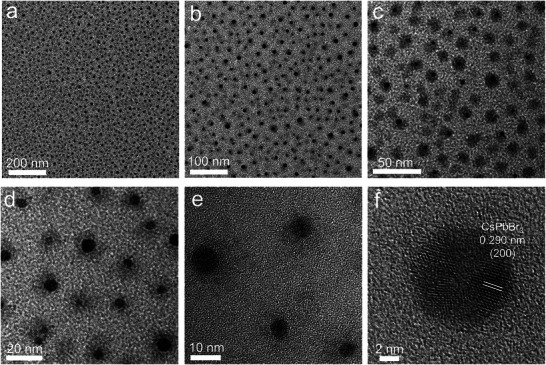
(a–f) TEM images of Na:CsPbBr_3_@CsPb_2_Br_5_ at different magnifications. The lattice fringes of CsPbBr_3_ were clearly observed in (f).

The TEM images of sample Na:CsPbBr_3_@CsPb_2_Br_5_ are shown in Figure 3. Very interesting, Na doped sample revealed different microstructure compared with Cu doped one. The Na doped sample revealed spherical morphology with uniform size and well dispersion in different magnifications (Figure 3a,b). The TEM images in Figure 3d,e further confirm that all spherical particles display typical core‐shell features, each containing only one distinct core. The HRTEM image in Figure 3f shows the core revealed high crystallinity, with a clear overall outline and a surrounding layer of lighter contrast at the edge. This contrast difference between the CsPbBr_3_ core and the CsPb_2_Br_5_ shell is consistent with the expected core–shell architecture, confirming the formation of a single‐core@shell structure. The measured lattice fringe spacing in the core region is 0.29 nm, corresponding to the (200) plane of cubic‐phase CsPbBr_3_ (Figure 3e,f). Particle size statistics (Figure S3) suggested an overall average size of 13.1 nm, and the average size of CsPbBr_3_ cores is 7.4 nm. Accordingly, the thickness of the CsPb_2_Br_5_ shell is estimated to be approximately 5.7 nm. In addition, the SEM image and element mappings (Figure S4) show the homogeneous distribution of element Cs, Pb, Br, and Na in the sample. The Na signals confirm the successful introduction of Na components. Because the ionic radius of Na is much smaller than that of Cs^+^, direct lattice substitution is unexpected. Therefore, Na^+^ was more likely incorporated via interstitial doping.

**FIGURE 4:**
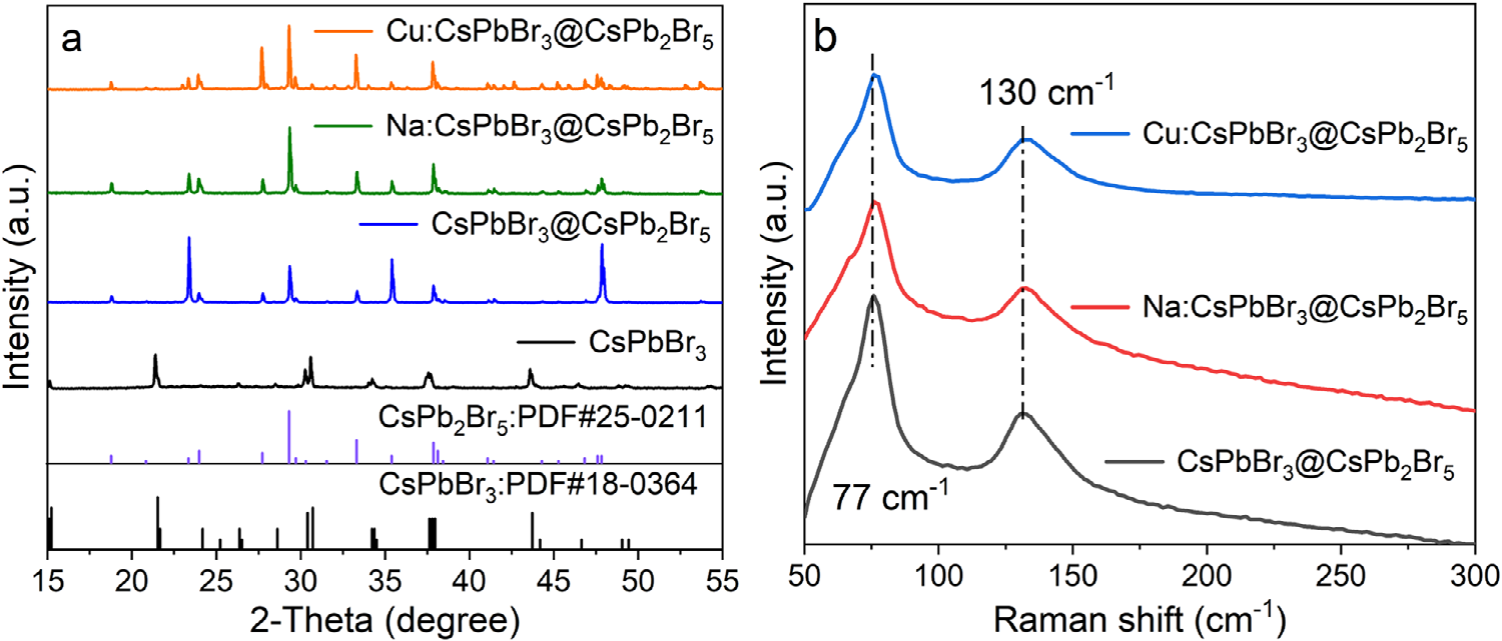
(a) XRD patterns of Na:CsPbBr_3_@CsPb_2_Br_5_ and Cu:CsPbBr_3_@CsPb_2_Br_5_. (b) Raman spectra of Na:CsPbBr_3_@CsPb_2_Br_5_ and Cu:CsPbBr_3_@CsPb_2_Br_5_.

To further investigate the crystal structure and local chemical environment, X‐ray diffraction (XRD) patterns and Raman spectroscopy were performed as presented in Figure 4. The XRD patterns (Figure 4a) show that the main XRD peaks of CsPbBr_3_ sample matched well with the standard reference pattern of monoclinic CsPbBr_3_ (PDF#18‐0364). In contrast, all water‐treated samples exhibit primary diffraction peaks that correspond closely to the CsPb_2_Br_5_ reference pattern (PDF#25‐0211), confirming the formation of a CsPb_2_Br_5_ shell. Notably, no distinct diffraction peaks attributable to the CsPbBr_3_ core are observed. This result indicated that Na or Cu doped CsPbBr_3_@CsPb_2_Br_5_ nanostructures is different from a traditional core‐shell structure as indiacted in TEM observation. This phenomenon is ascribed to the phase transfer process of CsPbBr_3_. As the continuous and well‐crystallized CsPb_2_Br_5_ shell constitutes the dominant volume fraction in the sample, its strong diffraction signal completely overshadows the weak contribution from the nanosized CsPbBr_3_ cores inside. Notably, neither Na nor Cu doped samples exhibit noticeable the peak shift compared with undoped sample, indicating that the doping did not significantly alter the crystal structure of CsPb_2_Br_5_. This result is consistent with the structural stability observed in the earlier morphological analysis, suggesting that both doping strategies introduce minimal disturbance to the periodic arrangement of the host lattice. However, combined with the lattice contraction (0.286 nm) observed in the core region of the Cu doped sample by HRTEM, the doping effect of Cu appears to be primarily localized within the CsPbBr_3_ core or at the interface, without producing a detectable overall peak shift in the XRD pattern.

Raman spectroscopy (Figure 4b) further elucidates the influence of doping on the local structure from the perspective of vibrational modes. All samples exhibit characteristic peaks at approximately 77 and 130 cm^−1^, corresponding to the external vibrational mode of the [PbBr_6_]^4−^ octahedra (associated with Cs^+^ motion) and the Pb‐Br stretching vibration, respectively [16]. The Raman peak positions of Na^+^ doped sample remain nearly identical to those of undoped sample, indicating that Na^+^ doping did not significantly alter the local bonding environment. In contrast, the characteristic peak near 130 cm^−1^ in Cu doped sample displays a distinct shift toward higher wavenumbers. This shift suggested increased vibrational frequency of Pb‐Br bonds, which typically corresponds to bond shortening or strengthening. These phenomena provide direct support for the incorporation of Cu^+^ in Pb^2+^ lattice sites, leading to enhanced local bonding and structural distortion. Consistent with the lattice contraction observed in the core region in TEM images, Raman analysis results demonstrated that the high sensitivity to local chemical environments compared with the result from XRD analysis, effectively capturing the subtle bonding modifications induced by Cu doping.

**FIGURE 5:**
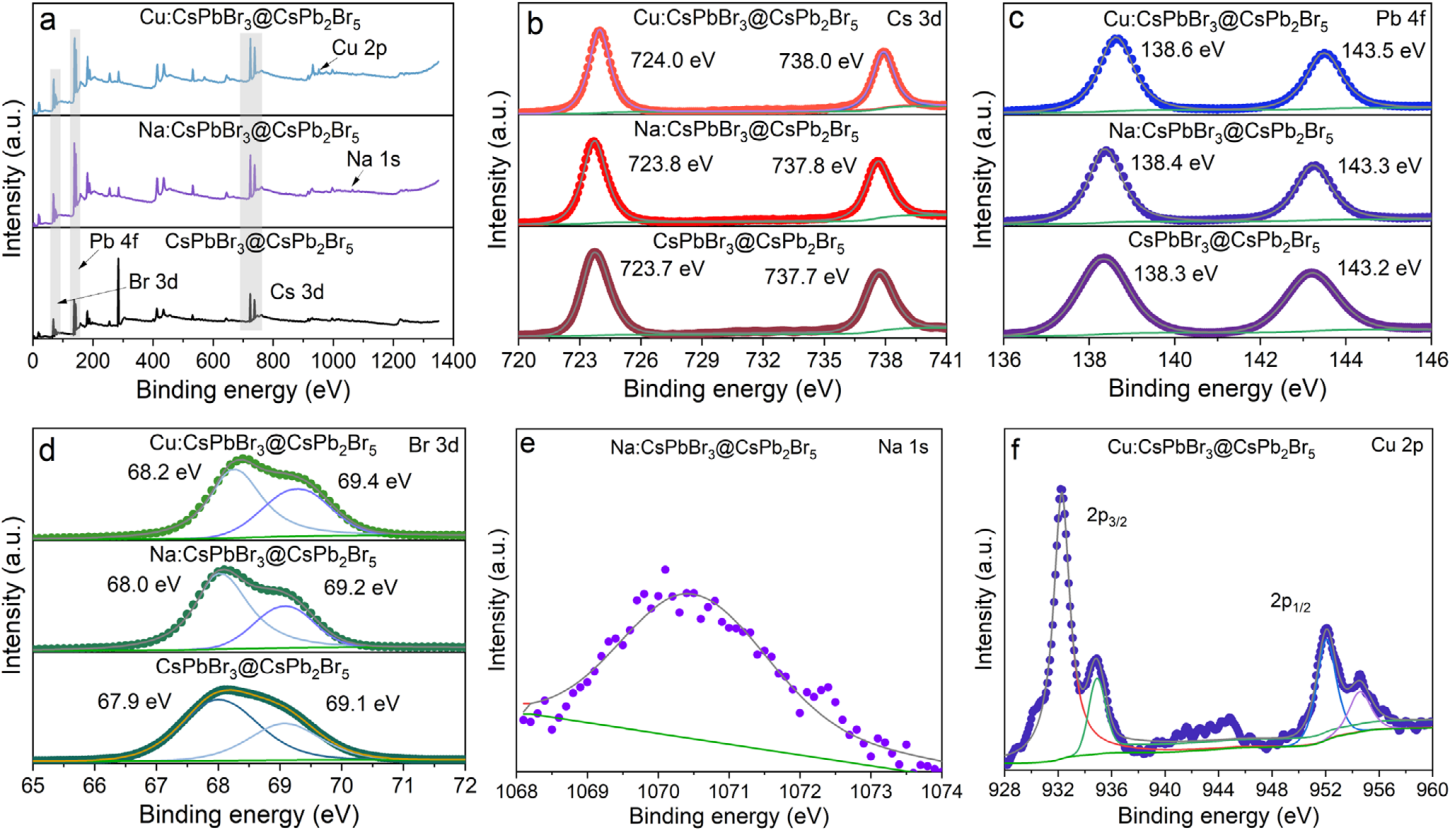
XPS spectra of samples: (a) Survey spectra, (b) Cs 3d spectra, (c) Pb 4f spectra, (d) Br 3d spectra, (e) Na 1s spectrum, (f) Cu 2p spectrum.

To further investigate Na^+^ and Cu^+^ doping and the effect on the electronic structure of composite nanostructures, X‐ray photoelectron spectroscopy (XPS) measurements were performed. The results are shown in Figure 5. The survey spectra (Figure 5a) verify the basic chemical composition of all samples, where characteristic peaks of Cs 3d, Pb 4f, and Br 3d are clearly identified. For the Na^+^ doped sample, a characteristic Na 1s signal is discernible in the survey spectrum, whereas the Cu^+^ doped sample clearly exhibits Cu 2p peaks, providing direct evidence for the successful incorporation of both elements. Further analysis of the high‐resolution spectra reveals that the Na 1s spectrum (Figure 5e) displays a symmetric single peak at 1070.5 eV. The Cu 2p fine spectrum (Figure 5f) shows two peaks located at 932.4 (Cu 2p_3/2_) and 952.3 eV (Cu 2p_1/2_), with a spin‐orbit splitting of 19.9 eV. Weak yet distinct satellite peaks characteristic of Cu^2+^ appear in the higher binding energy region (934.9  and 954.7 eV). These features confirm that copper primarily exists in the monovalent state (Cu^+^) within the material system, with a small fraction of Cu^+^ being oxidized to Cu^2+^ upon air exposure.

The XPS analysis results of Cs, Pb, and Br elements reveal notable changes in the electronic structure induced by doping. As shown in Figure 5b, the binding energy of Cs 3d_5/2_ peak revealed shift of 0.1 and 0.3 eV for Na and Cu doped samples, respectively. A similar trend was observed for Pb 4f_7/2_ spectra (Figure 5c), which moved to 138.4 eV   (Na^+^ doped) and 138.6 eV (Cu^+^ doped) from 138.3 eV. The most pronounced change appeared in Br 3d_5/2_ spectra (Figure 5d), which systematically shifted from 67.9  to 68.0  (Na^+^ doped) and 68.2 eV (Cu^+^ doped). These changes in binding energy indicated a reduction in electron cloud density around each atomic nucleus after doping. This effect can be attributed to electronic interactions between the doped ions and the host lattice. In particular, the partial substitution of Pb^2+^ by relatively electronegative Cu^+^ enhanced the attraction to the electron cloud of the coordinating Br^−^, thereby weakening the electron shielding effect of Br^−^ on neighboring Cs^+^ and Pb^2+^. Notably, the magnitude of the chemical shift induced by Cu^+^ doping is generally larger than that caused by Na^+^ doping. This observation aligns with the distinct doping mechanisms, Cu^+^ directly enters the B site (Pb^2+^ site) of the lattice, whereas Na^+^ likely occupies interstitial positions or resides at surface sites. This difference further elucidates the intrinsic driving force behind the formation of the unique “multi‑core@shell” structure observed in Cu^+^ doped sample. To directly study the possibility of Cu cluster formation, high‐angle annular dark‐field scanning transmission electron microscopy (HAADF‐STEM) images and energy dispersive spectroscopy (EDS) element mapping were performed on single Cu:CsPbBr_3_@CsPb_2_Br_5_. As shown in Figure S5, Cs, Pb, Br and Cu are uniformly distributed throughout the nanostructure without any detectable aggregation or separation. This uniform distribution provides direct evidence that Cu is incorporated into the CsPbBr_3_ lattice rather than forming a separate Cu‐rich cluster. Therefore, Cu^+^ is incorporated into the CsPbBr_3_ lattice in a substitutional status.

**FIGURE 6:**
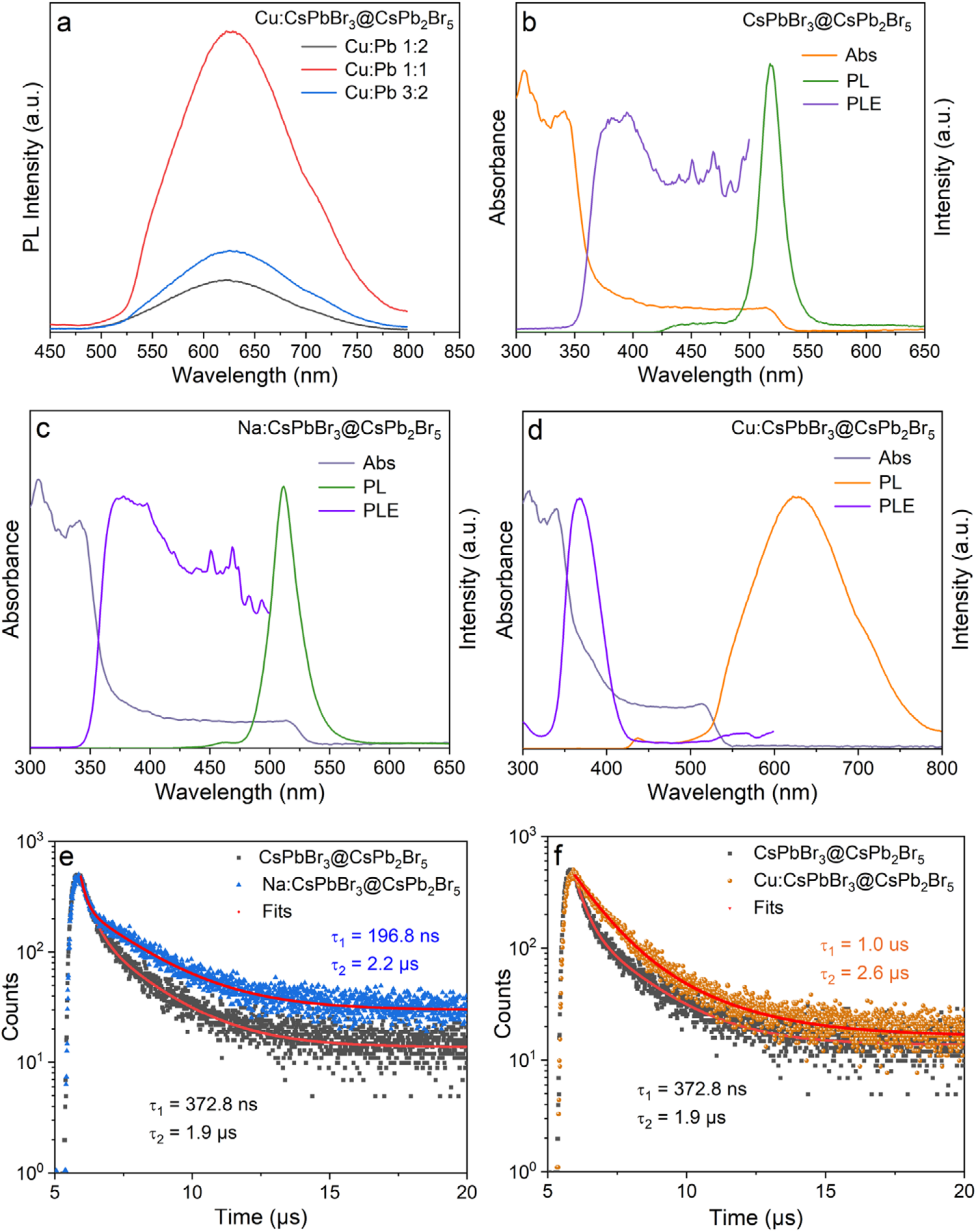
(a) PL spectra of Cu:CsPbBr_3_@CsPb_2_Br_5_ samples prepared using different Cu/Pb ratios. (b–d) DRS, PL, and PLE spectra of samples. (e and f) PL decay plots of samples.

Figure 6 shows the steady‐state and transient PL spectra of samples. Taking Cu:CsPbBr_3_@CsPb_2_Br_5_ as an example, the effect of Cu doping concentration on the PL behavior of CsPbBr_3_@CsPb_2_Br_5_ was first investigated by varying the molar ratios of CuBr to PbBr_2_ (Cu:Pb  =  1:2, 1:1, and 3:2) as shown in Figure 6a. Sample CsPbBr_3_@CsPb_2_Br_5_ exhibited a PL peak at 620 nm with a broad orange emission spectrum with a full width at half maximum (FWHM) of 138.1 as illustrated in Table S2, which is a typical STE emission. As Cu^+^ doping concentration increased, the PL peak position remained unchanged. Because the sample with a Cu:Pb ratio of 1:1 revealed the highest PL intensity, subsequent tests were performed using this composition. Notably, the luminescent performance of Cu‐doped samples differ strikingly from those of pure CsPbBr_3_@CsPb_2_Br_5_ and Na‐doped samples, as demonstrated in Figure 6b–d. Figure 6b presents the PL and PL excitation (PLE) spectra well as ultraviolet‐visible diffuse reflectance spectra (DRS) of pristine CsPbBr_3_@CsPb_2_Br_5_. The sample exhibited a sharp narrow PL peak at 517 nm with a FWHM of 21.8 nmand a PLQY of 55.4%, which is from CsPbBr_3_ NCs. The maximum excitation wavelength was 370 nm, and the PL peak position remained unchanged upon varying excitation wavelengths, indicating that the size of CsPbBr_3_ core is close to the exciton Bohr radius and thus experiences only weak quantum confinement effects. Moreover, the DRS shows two absorption edges located at 517  and 366 nm, which are attributed to the first excitonic absorption peaks of CsPbBr_3_ core and CsPb_2_Br_5_ shell, respectively [16]. The PL, PLE, and DRS of Na^+^ doped sample are displayed in Figure 6c. The Na^+^ doped sample revealed similar activities with those of pristine CsPbBr_3_@CsPb_2_Br_5_ sample, with a PL peak located at 516 nm with a FWHM of 22.6 nm. The peak position undergoes negligible shift or broadening, while the PLQY increased to 82.8%. The enhanceed PLQYs are ascribed to Na^+^ doping. Na^+^ acts as an efficient defect passivator, residing in interstitial positions or adsorbing on surfaces, thereby effectively passivating deep‐level defects inside the CsPbBr_3_ core as well as surface**/**interface defects.

Figure 6d displays the PL, PLE, and DRS spectra of a Cu doped sample. The DRS shows absorption features similar to those of the pristine CsPbBr_3_@CsPb_2_Br_5_, with absorption edges at 517  and 367 nm corresponding to the first excitonic absorption peaks of CsPbBr_3_ core and CsPb_2_Br_5_ shell, respectively. This indicates that Cu doping did not change the crystal structure of CsPbBr_3_@CsPb_2_Br_5_. However, the PL and PLE spectra of Cu^+^ doped sample exhibited clear difference from those of undoped sample. The PLE spectrum presents a symmetric peak centered at 367 nm, suggesting the presence of a single, efficient energy harvesting channel directed toward Cu^+^ emission centers. Meanwhile, the PL spectrum was located at 620 nm, showing a large Stokes shift relative to the absorption edge, which is characteristic of STE emission. The PLQY of the sample reaches to 45.6%.

To further confirm the uniformity of the luminescent centers, the PL spectra of samples under different excitation wavelengths were measured. The results show that as the excitation wavelength varies from 300 to 400 nm, the PL peak position remains consistently at 620 nm (Figure S6), and the spectral shape remains unchanged, suggesting the presence of one type of stable luminescent center in the sample, rather than a superposition of multiple centers. If copper clusters with different aggregation states existed in the system, different excitation wavelengths would selectively excite clusters of varying sizes or configurations, leading to notable changes in the PL peak position or spectral profile. Therefore, these results provide an alternative perspective to exclude Cu clusters as the primary luminescence source, further supporting the mechanism of self‐trapped exciton emission induced by Cu^+^ substitutional doping.

Additionally, Zn^2+^ was employed as a dopant to prepare Zn:CsPbBr_3_@CsPb_2_Br_5_. The results demonstrate that the substitution of Pb^2+^ by Zn^2+^ also introduces strong local lattice distortion, significantly enhancing electronphonon coupling and consequently inducing a stable STE state within the CsPbBr_3_ bandgap (Figure S7). To further investigate the STE luminescent properties, Cu:CsPb(Cl/Br)_3_@CsPb_2_(Cl/Br)_5_ samples were prepared. The results indicate that although the incorporation of Cl**^−^** resulted in a pronounced blue shift of the absorption edge (Figure S8), the incorporation of Cl^−^ results in a slight blue shift of the STE PL peak from 627 to 623 nm. The magnitude of the STE peak shift (4 nm) is small, indicating that the STE energy level is mainly determined by the local lattice distortion caused by Cu^+^ substitution, while the overall halide composition has a secondary effect. In Cu^+^‐based host materials such as Cs_3_Cu_2_I_5_ and CsCu_2_I_3_, Cu^+^ intrinsic emission occurs at 445 or 568 nm [38, 39], distinct from our observed 620 nm. HRTEM and Raman analysis confirm that Cu^+^ substitutes Pb^2+^ in the CsPbBr_3_ lattice, inducing lattice distortion without forming a separate Cu^+^‐containing phase. Moreover, the microsecond‐scale lifetime and broad bandwidth are characteristic of STE, whereas intrinsic Cu^+^ emission typically exhibits shorter lifetimes and narrower bandwidths. The similar STE emission in Cu^+^ and Zn^2+^ doped samples can be rationalized by recent findings that broadband emission in halide perovskites is governed by inter‐octahedron distortion (Pb displacement) rather than local octahedron geometry or chemical composition [36]. Both dopants induce comparable lattice distortion, creating similar STE centers. In Cu:CsPb(Cl/Br)_3_@CsPb_2_(Cl/Br)_5_, the STE emission is determined by the lattice distortion induced by Cu^+^ substitution, independent of the overall halide composition.

To get deep insight into the fundamental influence of different doping strategies on the luminescence dynamics of the materials, the time resolved PL decay curves of samples CsPbBr_3_@CsPb_2_Br_5_, Na:CsPbBr_3_@CsPb_2_Br_5_, and Cu:CsPbBr_3_@CsPb_2_Br_5_ were measured and shown in Figure 6e,f. The corresponding fitting parameters were summarized in Table S3. The decay kinetics exhibit marked differences, revealing distinct operational mechanisms for Na^+^ and Cu^+^ dopants. The PL decay plots of the pristine CsPbBr_3_@CsPb_2_Br_5_ sample was fitted with a biexponential function [32], which yielded two lifetime components: shorter lifetime component τ_1_ of 372.8 ns (30.8%) and longer lifetime component τ_2_ of 1925.5 ns (69.2%). The average lifetime (τ_ave_) was 1802.2 ns. The shorter component τ_1_ is generally associated with non‐radiative recombination channels caused by incompletely passivated surface or interface defects, whereas the longer component τ_2_ primarily arises from the intrinsic radiative recombination of excitons within the CsPbBr_3_ core. A CsPb_2_Br_5_ shell provided fundamental carrier confinement and surface passivation, rendering the radiative recombination pathway dominant.The introduction of Na^+^ led to a targeted optimization of the decay kinetics.

For sample Na:CsPbBr_3_@CsPb_2_Br_5_, the short lifetime component τ_1_ decreases significantly to 196.8 ns, and its amplitude contribution drops sharply to 9.1%. Concurrently, the long lifetime component τ_2_ increases to 2.18 µs with its contribution rising substantially to 90.9%, resulting in an increased τ_ave_ of 2.16 µs. This evolution clearly demonstrates that Na^+^ effectively passivates defects and decreased non‐radiative recombination centers, such as halogen vacancies, within the CsPbBr_3_ core and at the core‐shell interface. Consequently, the probability of photogenerated carriers being trapped and quenched by defects is greatly reduced, promoting more efficient recombination via the intrinsic radiative channel. Therefore, the primary role of Na^+^ doping lies in defect engineering to optimize the environment of the original excitonic emission pathway, suppress nonradiative losses, and thereby enhance the PLQYs. In contrast, the decay kinetics of sample Cu:CsPbBr_3_@CsPb_2_Br_5_ undergo a fundamental transformation. Although the PL decay plots still requires biexponential fitting, both lifetime components reach the microsecond regime, with τ_1_ = 1006.3 ns (49.6%) and τ_2_ = 2608.9 ns (50.4%), yielding anaverage lifetime τ_ave_ of 2167.7 ns. This behavior is attributed to the successful establishment of localized emissive energy levels by Cu^+^ within the CsPbBr_3_ lattice. The two longlived components in the PL decay correspond to the STE emission induced by Cu^+^ in the localized environment. These results indicate that Cu^+^ doping does not merely passivate defects; rather, it reconstructs an entirely new radiative recombination channel centered on Cu^+^. While Na^+^ enhanced the efficiency of the original bandedge excitonic emission by strongly passivating defects, Cu^+^ fundamentally alters the luminescence nature of the material by introducing a novel, longlived emissive center.

**FIGURE 7:**
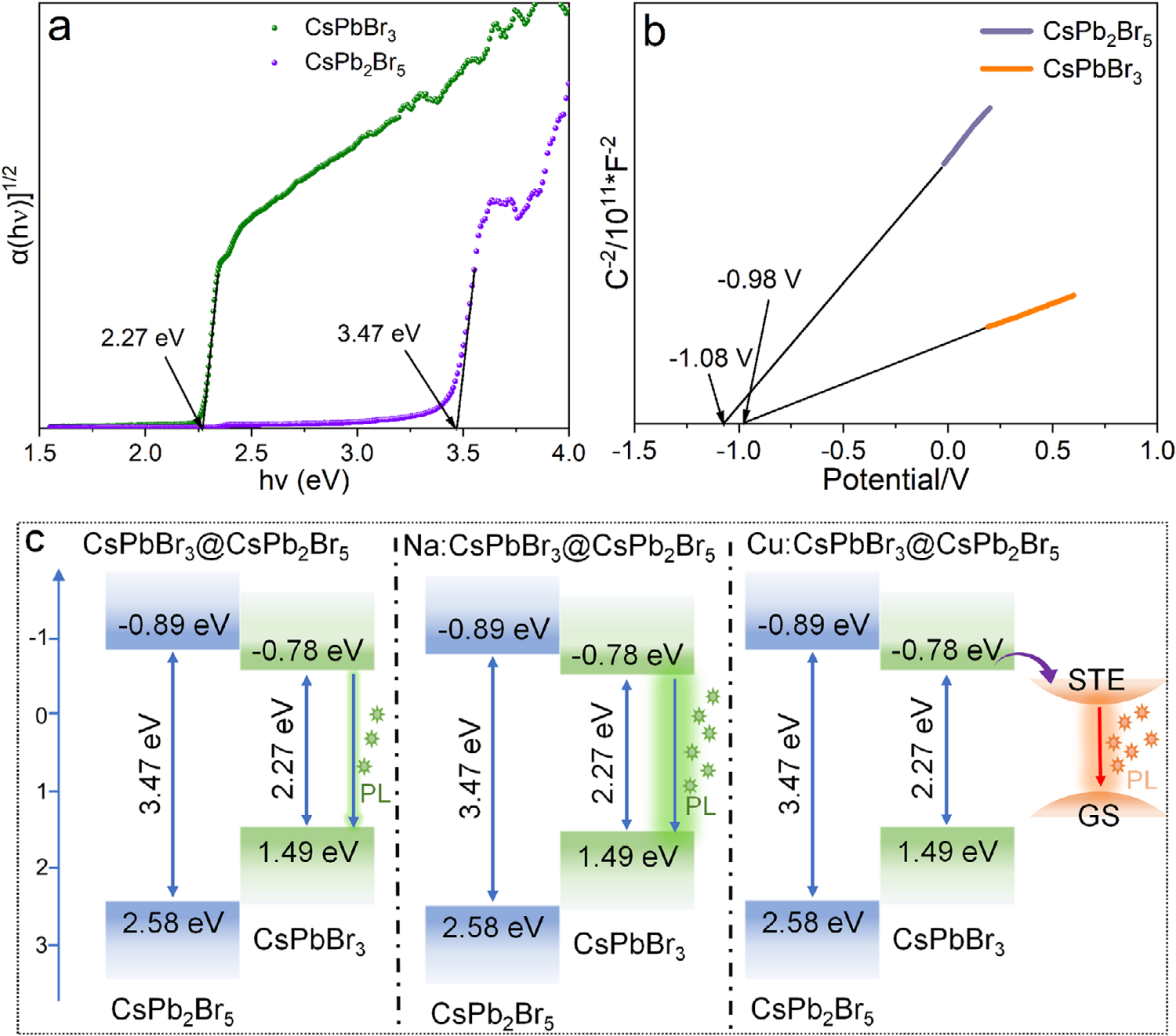
(a) UV–vis DRS of Cu:CsPbBr_3_ and Cu:CsPbBr_3_@CsPb_2_Br_5_ samples. (b) Tauc plots of Cu:CsPbBr_3_ and Cu:CsPbBr_3_@CsPb_2_Br_5_ samples. (c) Band gap scheme of samples.

The PLQY difference between Na‐doped (82.8%) and Cu‐doped (45.6%) samples reflects distinct recombination pathways. For Na:CsPbBr_3_@CsPb_2_Br_5_, Na^+^ occupies interstitial sites, passivating deep‐level defects and suppressing nonradiative recombination, as evidenced by the PL decay kinetics where the short‐lived component decreases from 30.8% to 9.1% upon doping. In contrast for Cu:CsPbBr_3_@CsPb_2_Br_5_, Cu^+^ substitution induces lattice distortion, creating STE centers that introduce a new radiative channel. However, the multi‐core@shell structure and lattice strain likely introduce additional nonradiative pathways, resulting in a lower PLQY (45.6%). This is consistent with the larger short‐lived component (49.6%) and longer average lifetime (2167.7 ns) compared to the Na‐doped sample. To elucidate the PL mechanism of Cu:CsPbBr_3_@CsPb_2_Br_5_ and the doped derivatives, the band gap activities of samples are shown in Figure 7. The DRS spectra (Figure S9) and Tauc plots (Figure 7a) obtained from the DRS spectra indicate that CsPb_2_Br_5_ possesses a band gap of 3.47 eV, which is larger than that of CsPbBr_3_ (2.27 eV). The obtained band gaps are consistent with literature values [40]. Subsequently, the flat‐band potentials of CsPbBr_3_ and CsPb_2_Br_5_ were determined from Mott‐Schottky plots to be −0.98  and −1.08 V, respectively, as shown in Figure 7c,d [33, 34, 35]. The conduction band (CB) potentials of samples were estimated using the standard hydrogen electrode Equation (1) as follows:

(1) $$E_{\mathrm{NHE}}=E_{\mathrm{Ag}/\mathrm{AgCI}}+0.059*pH+0.197$$

where, the CB potentials of CsPbBr_3_ and CsPb_2_Br_5_ are located at −0.78  and −0.89 eV, respectively. According to their band gaps, the valence band (VB) potentials are calculated to be 1.49 eV for CsPbBr_3_ and 2.58 eV for CsPb_2_Br_5_. As illustrated in the energy‐level diagram in Figure 7c, both the CB minimum and the VB maximum of CsPb_2_Br_5_ are higher than those of CsPbBr_3_, forming a typical type‐I band alignment, resulting in efficient transfer of excitation energy from the CsPb_2_Br_5_ shell to the CsPbBr_3_ core upon photon absorption, establishing CsPb_2_Br_5_ as the energy donor and CsPbBr_3_ as the luminescent acceptor. For the undoped CsPbBr_3_@CsPb_2_Br_5_ and Na:CsPbBr_3_@CsPb_2_Br_5_ samples, the PL is predominantly from the intrinsic band edge exciton recombination within the CsPbBr_3_ core. Na^+^ acts as an efficient defect passivator, residing in interstitial sites, surface adsorbed positions, or grain boundary regions. It effectively passivated deep‐level defects inside the CsPbBr_3_ core as well as surface/interface defects states, without introducing new luminescent energy levels within the band gap. The PL retains the characteristic excitonic radiative transition of CsPbBr_3_ NCs.

In contrast, sample Cu:CsPbBr_3_@CsPb_2_Br_5_ exhibited markedly different luminescent behavior. Cu^+^ instead of Pb^2+^ introduced significantly local lattice strain and distortion. This distortion strongly enhanced the local electron‐phonon coupling around Cu^+^ sites [36], creating ideal conditions for the formation of STE, as illustrated in Figure 7c. This mechanism shares a fundamental physical basis with strategies used in rare earth metal halides, where dopants such as Bi^3+^ or Sb^3+^ are employed to induce STE emission [37]. In this model, after energy transfer from CsPb_2_Br_5_ to CsPbBr_3_, the photogenerated carriers are no longer predominantly recombined via band‐edge pathways. Instead, they are effectively captured by the localized STE states induced by Cu^+^, followed by radiative recombination from the STE state to the ground state, yielding the observed PL. The microsecond‐scale lifetime component is one of the characteristic features of radiative recombination of STEs. However, in the core‐shell structure of current samples, such lifetime is also influenced by carrier confinement effects. Therefore, we mainly rely on the broad emission spectrum, the large Stokes shift, and the excitation‐wavelength‐independent emission peak position to confirm the STE mechanism, while using the lifetime data as supportive evidence. The luminescent process of CsPbBr_3_@CsPb_2_Br_5_ nanostructures is centered on energy transfer from CsPb_2_Br_5_ to CsPbBr_3_, while the specific emission pathway is precisely regulated by the dopant ions. Undoped and Na‐doped samples exhibit intrinsic band‐edge exciton emission of CsPbBr_3_, whereas the Cu‐doped sample mediated emission via Cu^+^ induced STE states, achieving a mechanistic transition from excitonic emission to localized impurity state emission.

Representative STE‐active materials are compared in Table 1. Cs_3_Cu_2_I_5_, featuring a 0D structure, exhibits efficient blue STE emission at 445 nm with a high PLQY of 91.2% [38]. In contrast, CsCu_2_I_3_, which possesses a 1D chain‐like structure, shows yellow emission at 568 nm with a PLQY of 84.8% [39]. Mn^2^^+^ doping introduces a new emission center at 564 nm in Cs_3_Cu_2_I_5_, achieving a high radio luminescence yield; however, its PLQY of 38.3% is lower than that of our system [40]. In addition, Er^3+^, Bi^3+^ co‐doped Cs_2_AgInCl_6_ double perovskite enables efficient near‐infrared emission but requires a moderate synthesis temperature and exhibits only moderate room‐temperature stability [41]. In this work, the CsPbBr_3_@CsPb_2_Br_5_ sample features a CsPb_2_Br_5_ shell that provides exceptional protection against moisture, ultraviolet irradiation, and water exposure, a level of stability rarely achieved in previously reported STE‐active systems. The combination of Cu dopant‐induced STE emission and the enhanced stability afforded by the core–shell structure endows CsPbBr_3_@CsPb_2_Br_5_ with significant application potential.

**TABLE 1:** Comparison of STE emission properties in representative metal halide systems.

Sample	PL peak [nm]	PLQY [%]	Stability	Synthesis	Refs.
Sb^3+^:Cs_2_NaInCl_6_	580	78.9	Moderate	Hydrothermal	[23]
Cs_3_Cu_2_I_5_	445	91.2	Good	Solid‐state reaction	[38]
CsCu_2_I_3_	568	84.8	Good	Vacuum thermal evaporation	[39]
Cs_3_Cu_2_I_5_:Mn^2+^	564	38.3	Excellent	Solid‐state	[41]
Cs_2_AgInCl_6_:Bi^3+^,Er^3+^	1540	/	Moderate	Hydrothermal	[42]
Cu:CsPbBr_3_@CsPb_2_Br_5_	620	45.6	Excellent	Aqueous	This work

**FIGURE 8:**
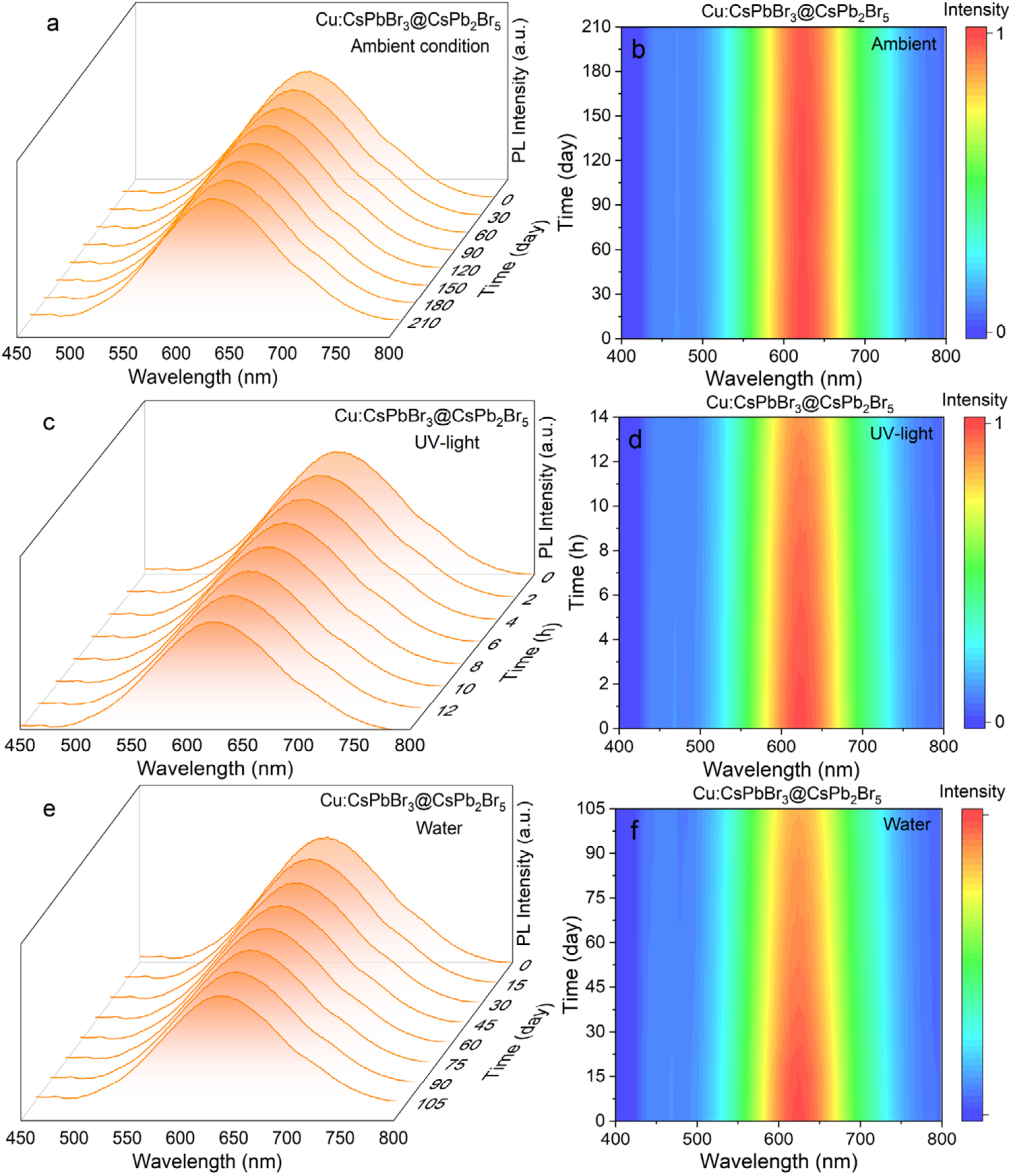
PL stability test of the Cu:CsPbBr_3_@CsPb_2_Br_5_. (a) and (b) Ambient condition, (c) and (d) UV‐light irradiation, (e) and (f) water treatment.

Based on systematic tests of the PL stability of the Cu:CsPbBr_3_@CsPb_2_Br_5_ sample (Figure 8), the material demonstrates excellent long‐term stability under various harsh conditions. As shown in Figure 8a,b, the PL intensity of the sample was retained more than 90% of the initial value after storage for 105 days under ambient conditions while the PL peak position was remained unchanged, suggesting a high stability. Under continuous ultraviolet irradiation (λ_ex_  =  365 nm) for 12 h, the PL intensity of the sample was maintained over 95% of the initial value as shown in Figure 8c,d.

In addition, the sample also shows excellent tolerance in aqueous solutions. After direct immersion in water for 105 days, bright PL was observed and the PL spectra did not shifted, indicating extraordinary stability compared with traditional hydrophobic CsPbBr_3_ NCs as shown in Figure 8e,f. The stability of the luminescent properties also proves that the crystal structure and core‐shell structure remain intact, because the structural degradation will cause PL quenching or spectral shift. The comprehensive stability can be primarily attributed to the effective physical and chemical barrier provided by the CsPb_2_Br_5_ shell. This shell with wide band gap can physically block the direct penetration of water molecules and oxygen, thereby mitigating the erosion of the luminescent core. Furthermore, it effectively suppresses ion migration, particularly the surface migration and leaching of Cs^+^ and halide ions, which helps maintain the integrity of the core‐shell interface. In addition, the shell passivates surface defects, reducing environmentally induced non‐radiative recombination channels. Together, these effects collectively enhance the overall stability of the core‐shell structure. The stability of the Na:CsPbBr_3_@CsPb_2_Br_5_ sample was also evaluated, as shown in Figure S10. The results confirm that this sample similarly exhibits excellent stability under various harsh conditions. The outstanding PL stability of the samples under ambient, light‐exposure, and aqueous conditions mainly originates from the encapsulation and protective effects provided by the CsPb_2_Br_5_ shell. This offers a crucial material foundation for their application in optoelectronic devices requiring long‐term reliable operation.

**FIGURE 9:**
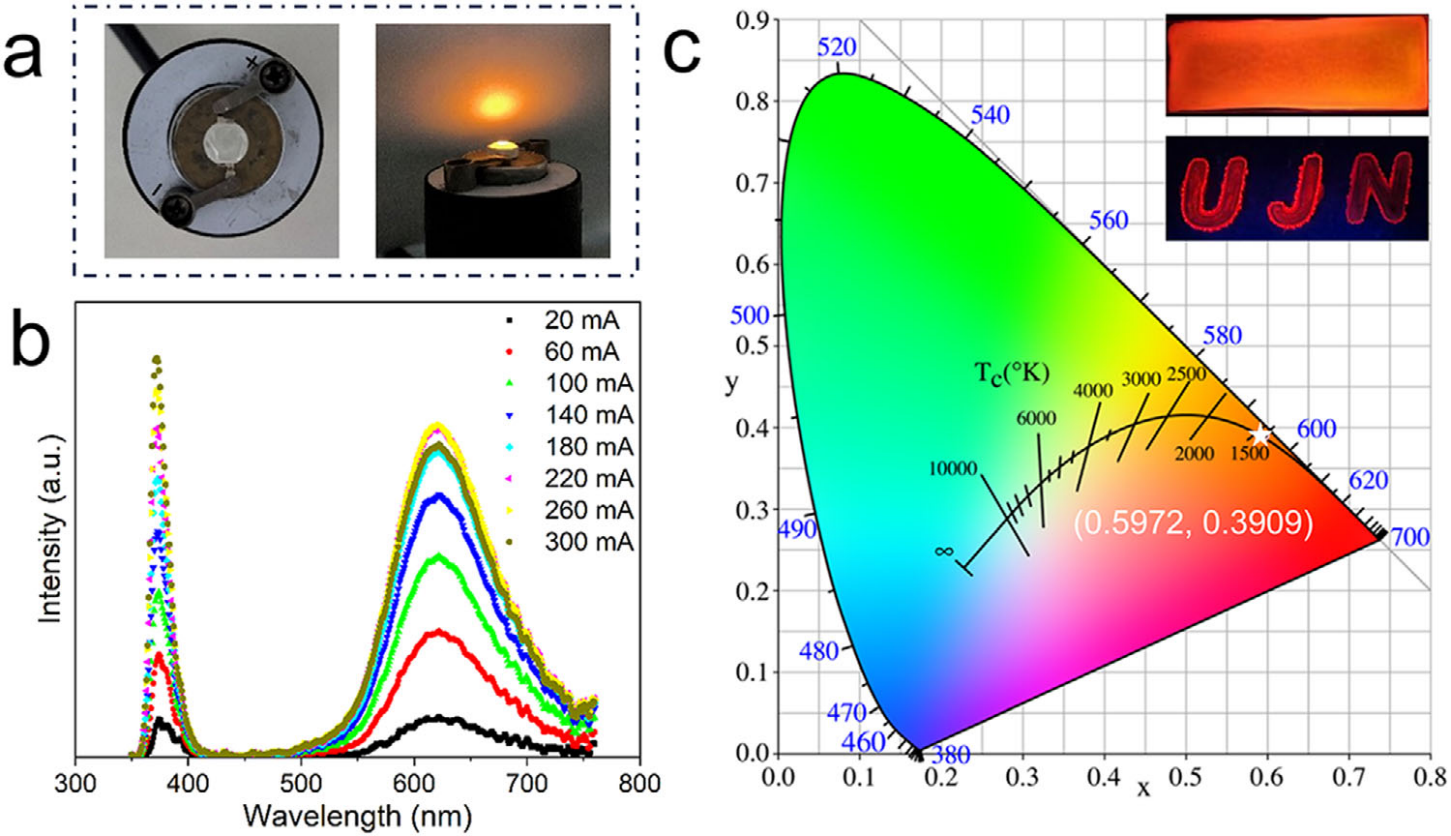
(a) Photos of LED devices in natural light and working conditions. (b) PL spectra of LED devices under different operating currents. (c) CIE chromaticity diagram of the LED device coated with Cu:CsPbBr_3_@CsPb_2_Br_5_ phosphor. The insets in (c) show the pictures of luminescent films and UJN patterns prepared using sample Cu:CsPbBr_3_@CsPb_2_Br_5_.

To verify the practical application potential of sample Cu:CsPbBr_3_@CsPb_2_Br_5_, LED devices were prepared using Cu:CsPbBr_3_@CsPb_2_Br_5_ nanostructures as a light‐emitting layer on a 365 nm LED chip. As shown in Figure 9a, the device emits bright orange light during operation. The PL spectra measured under different driving currents (Figure 9b) reveal broad emission centered at 620 nm even at a low current of 20 mA. As the current increases, the intensity of the main peak grows significantly and becomes dominant, indicating that the STE‐dominated recombination channel exhibits higher efficiency under high carrier injection. Throughout the current increase up to 300 mA, both the peak position and spectral profile of the PL remain highly stable, with no noticeable peak shift or spectral broadening, demonstrating excellent electrical and optical stability. Further chromaticity analysis (Figure 9c) shows that the device exhibited color coordinates of (0.60, 0.39), a color rendering index of 62, and a luminous efficacy of 1.02 lm/W. The calculated correlated color temperature is approximately 1430 K, which falls within the typical range of warm orange light and is suitable for comfortable indoor lighting. Therefore, the LED device incorporating Cu:CsPbBr_3_@CsPb_2_Br_5_ nanostructures as the emitter revealed stable PL, an appropriate warm orange‐red color, and good reproducibility, thereby preliminarily validating the material potential for applications in solid‐state lighting.

## 3 Conclusions

CsPbBr_3_@CsPb_2_Br_5_ core‐shell nanostructures were created by the dissolution and phase transfer of hydrophilic CsPbBr_3_ using a two‐step synthesis. The nanostructures revealed controllable, bright, and high stable PL because of Na^+^ and Cu^+^ doping. The experimental results indicated that interstitial doping with Na^+^ significantly passivated deep‐level defects, thereby improving the PLQY of the green luminescence of CsPbBr_3_. In contrast, lattice substitution with Cu^+^ induced local structural distortion, successfully establishing STE luminescent centers and realizing a mechanistic transition from narrow‐band green to broad orange‐red emission. Moreover, the CsPb_2_Br_5_ shell effectively enhanced the environmental tolerance and long‐term stability, laying a foundation for its practical application. An LED device fabricated using Cu^+^ doped nanostructures exhibited stable orange‐red PL, preliminarily confirming the potential in solid‐state lighting field. These results provide an effective synergistic strategy to construct core‐shell nanostructures and study novel PL phenomenon of perovskite nanomaterials. In addition, the deep discussion between the PL performance and microstructure CsPbBr_3_@CsPb_2_Br_5_ core‐shell nanostructures resulted in the understanding of STE‐dominated recombination to create novel luminescent materials. Cu:CsPbBr_3_@CsPb_2_Br_5_ material features exceptional water/UV stability, broadband STE emission for warm‐color lighting, and a scalable aqueous synthesis route. Although the current LED devices show moderate performance indicators, further optimization of the device structure is expected to improve efficiency and operational stability.

## 4 Experimental Section

### 4.1 Chemicals

Cesium bromide (CsBr, 99.9%), cesium chloride (CsCl, 99.9%), lead bromide (PbBr_2_, 99%), and zinc bromide (ZnBr_2_, 99.9%) were purchased from Shanghai Aladdin Biochemical Technology Co. Ltd. Sodium bromide (NaBr, 99.0%) were purchased from Sinopharm Chemical Reagent Co., Ltd. Cuprous bromide (CuBr, 99.0%) and hydrobromic acid (HBr, 40%) were purchased from Macklin Biochemical Technology Co. Ltd.

### 4.2 Synthesis of CsPbBr_3_

Monoclinic CsPbBr_3_ powders were synthesized via a co‐precipitation technique in an ice‐water bath. In particularly, precursor solution 1 was prepared by dissolving CsBr (1 mmol, 0.213 g) in 0.5 mL of water. Meanwhile, precursor solution 2 was obtained by dissolving PbBr_2_ (1 mmol, 0.367 g) in 2 mL of HBr. Solution 2 was then placed in an ice water bath. Under continuous vigorous stirring, solution 1 was dropwise into solution 2. Due to the solubility differences, the ionic concentration product of Cs^+^, Pb^2+^, and Br^−^ exceeded the solubility of CsPbBr_3_ product, triggering rapid precipitation. The resulting orange yellow precipitates were isolated by centrifugation, washed for three times with ethanol, and dried naturally in dark to get resulting CsPbBr_3_ powder sample.

### 4.3 Synthesis of CsPbBr_3_@CsPb_2_Br_5_

As prepared CsPbBr_3_ powders were dispersed in 2 mL of deionized water and sonicated for 5 min until the color of the powder completely changed from orange‐yellow to white, indicating the phase transformation to a core‐shell structure. The resulting precipitates were collected by centrifugation, dried naturally in the dark, and finally obtained as CsPbBr_3_@CsPb_2_Br_5_ powders.

### 4.4 Synthesis of Na or Cu doped CsPbBr_3_@CsPb_2_Br_5_ Nanostructures

The synthesis of Na:CsPbBr_3_@CsPb_2_Br_5_ was finished followed a same procedure as described above for preparation of CsPbBr_3_ and CsPbBr_3_@CsPb_2_Br_5_, except for NaBr addition in precursor solution 1. As for sample Cu:CsPbBr_3_@CsPb_2_Br_5_, CuBr was added in precursor solution 2 and other parameters are same with sample CsPbBr_3_@CsPb_2_Br_5_.

### 4.5 Fabrication of LED

Cu:CsPbBr_3_@CsPb_2_Br_5_ sample was uniformly mixed with silicone gel at a mass ratio of 2:1. The resulting mixture was then encapsulated onto a blue LED chip (365 nm) to fabricate a simple prototype LED device.

### 4.6 Characterization

A comprehensive series of characterizations were performed to analyze the samples. Crystal structures were determined using XRD diffractometer (Rigaku SmartLab SE). Microstructural information was obtained via Raman spectroscopy (LabRAM HR). Chemical composition and elemental states were examined by XPS (Thermo Scientific K‐Alpha). The morphology and microstructure were observed by HRTEM (JEOL JEM‐2100F). Elemental distribution was analyzed using SEM (JSM‐7800F) equipped with energy‐dispersive X‐ray spectroscopy. Optical properties were evaluated by steady–state PL spectra and DRS under 365 nm light excitation, recorded on a Hitachi F‐4600 spectrofluorometer and a Hitachi U‐4100 spectrophotometer, respectively. Carrier recombination dynamics were studied by measuring time‐resolved PL decay plots (λ_ex_ = 370 nm) using a JY‐IBH fluorescence lifetime spectrometer. The relative PLQY was quantified with rhodamine 6G (a PLQY of 95% in ethanol) as the reference. The luminescent performance of as‐fabricated LED devices was assessed using a multifunctional optical flux tester (OHSP‐350 M) to measure parameters such as luminous flux, chromaticity coordinates, and correlated color temperature. Mott‐Schottky measurements were performed using a three‐electrode system in 0.1 M Na_2_SO_4_ aqueous solution, with the sample‐loaded ITO glass as the working electrode, Ag/AgCl as the reference electrode, and a Pt plate as the counter electrode. The measurement frequency was set to 1000 Hz.

## Conflicts of Interest

The authors declare no conflicts of interest.

## Supporting information




**Supporting File**: smll73956‐sup‐0001‐SuppMat.docx.

## Data Availability

The data that support the findings of this study are available on request from the corresponding author. The data are not publicly available due to privacy or ethical restrictions.
