# Supplementary material for: Bright Self‐Trapped Exciton Emission of CsPbBr3@CsPb2Br5 Nanostructures Created by Dissolution and Recrystallization of Hydrophilic Cu:CsPbBr3
Source: Small. 2026 May 26;22(39):e73956. doi: 10.1002/smll.73956 (PMC13360731; doi:10.1002/smll.73956)
Supplement: Supplementary file 1 — Supporting File: smll73956‐sup‐0001‐SuppMat.docx. [file SMLL-22-e73956-s001.docx]

Supporting Information

Bright Self-Trapped Exciton Emission of CsPbBr_3_@CsPb_2_Br_5_ Nanostructures Created by Dissolution and Recrystallization of hydrophilic Cu:CsPbBr_3_

Wenbin Shi, Xiao Zhang,* Hsueh Shih Chen, San Ping Jiang, Ping Yang*

((Optional Dedication))

Dr. W. Shi, Prof. P. Yang

School of Material Science and Engineering, University of Jinan, Jinan, 250022, PR China
E-mail: mse_yangp@ujn.edu.cn, orcid.org/0000-0002-1061-3383

Prof. X. Zhang

Advanced Institute for Materials Research (WPI-AIMR), Tohoku University, Sendai, 980-8577, Japan.

E-mail: zhang.xiao.d8@tohoku.ac.jp, orcid.org/0000-0003-2547-7262

Prof. H. S. Chen

Department of Materials Science and Engineering, National Tsing Hua University, Hsinchu City, 300, Taiwan

Prof. S. P. Jiang

WA School of Mines: Minerals, Energy and Chemical Engineering, Curtin University, Perth, WA6845, Australia

**Table 1** Preparation conditions of samples.

| Sample | Doping ions (X) | (X):Pb | T (°C) |
| --- | --- | --- | --- |
| CsPbBr_3_@CsPb_2_Br_5_ | n/a | n/a | 25 |
| Na:CsPbBr_3_@CsPb_2_Br_5_ | Na | 1:1 | 25 |
| Cu:CsPbBr_3_@CsPb_2_Br_5_ | Cu | 1:2 | 25 |
| Zn:CsPbBr_3_@CsPb_2_Br_5_ | Zn | 1:2 | 25 |
| Cu:CsPb(Cl/Br)_3_@CsPb_2_(Cl/Br)_5_ | Cu | 1:2 | 25 |

**Table S2** PLQY, PL peak wavelength, FWHM of PL spectra of samples.

| Sample | PLQY (%) | PL peak (nm) | FWHM (nm) |
| --- | --- | --- | --- |
| CsPbBr_3_@CsPb_2_Br_5_ | 55.4 | 517 | 21.8 |
| Na:CsPbBr_3_@CsPb_2_Br_5_ | 82.8 | 516 | 22.6 |
| Cu:CsPbBr_3_@CsPb_2_Br_5_ | 45.6 | 627 | 138.1 |
| Zn:CsPbBr_3_@CsPb_2_Br_5_ | 43.1 | 624 | 143.9 |
| Cu:CsPb(Cl/Br)_3_@CsPb_2_(Cl/Br)_5_ | 40.5 | 623 | 136.9 |

**Table S3** Components B_1_ and B_2_, time constants τ_1_,τ_2_, and average lifetime τ_ave_ of samples CsPbBr_3_@CsPb_2_Br_5_, Na:CsPbBr_3_@CsPb_2_Br_5,_ and Cu:CsPbBr_3_@CsPb_2_Br_5_.

| Sample | τ_1_ (ns) | B_1_ (%) | τ_2_ (ns) | B_2_ (%) | τ_ave_ (ns) |
| --- | --- | --- | --- | --- | --- |
| CsPbBr_3_@CsPb_2_Br_5_ | 372.8 | 30.8 | 1925.5 | 69.2 | 1802.2 |
| Na:CsPbBr_3_@CsPb_2_Br_5_ | 196.8 | 9.1 | 2175.5 | 90.9 | 2157.7 |
| Cu:CsPbBr_3_@CsPb_2_Br_5_ | 1006.3 | 49.6 | 2608.9 | 50.4 | 2167.7 |


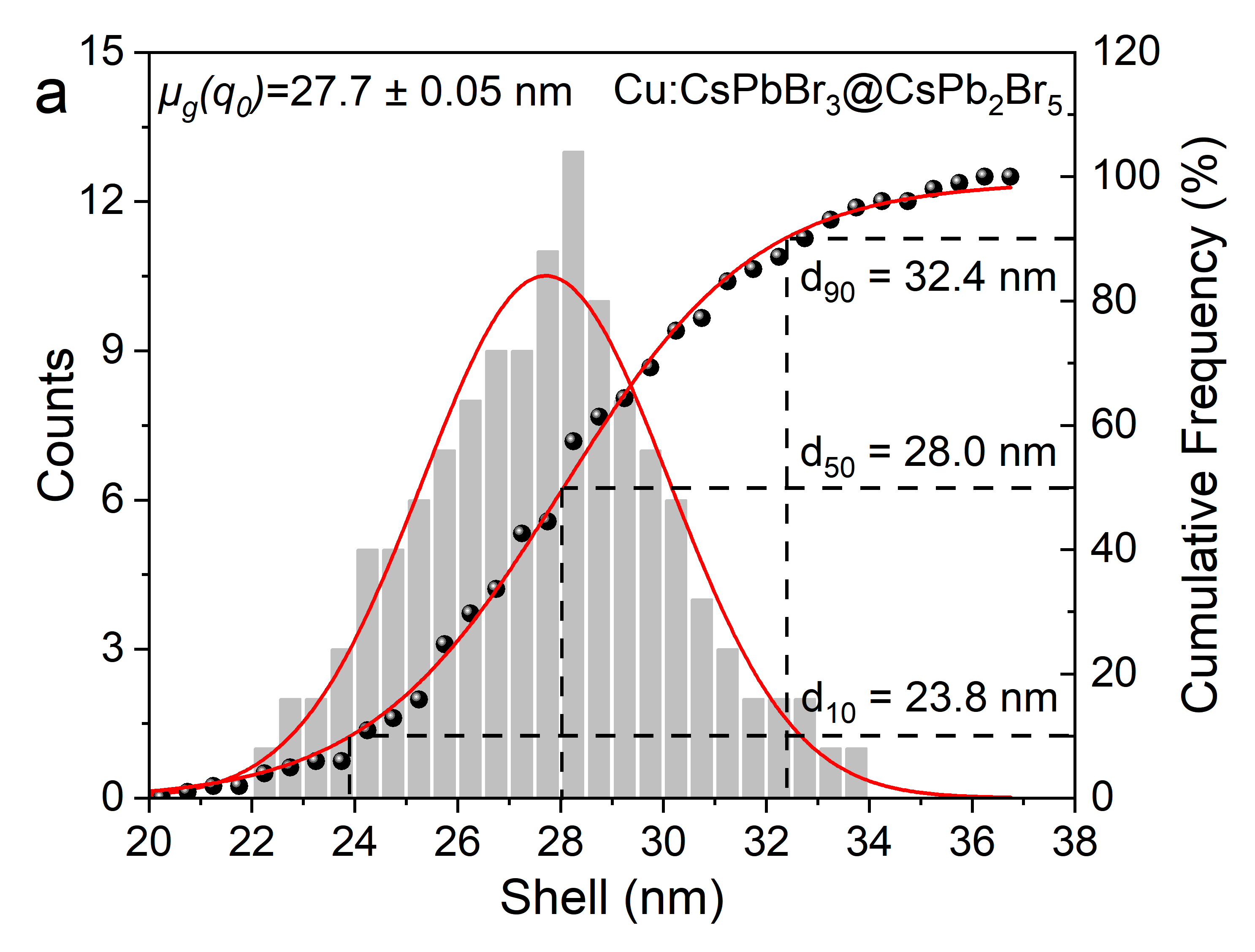

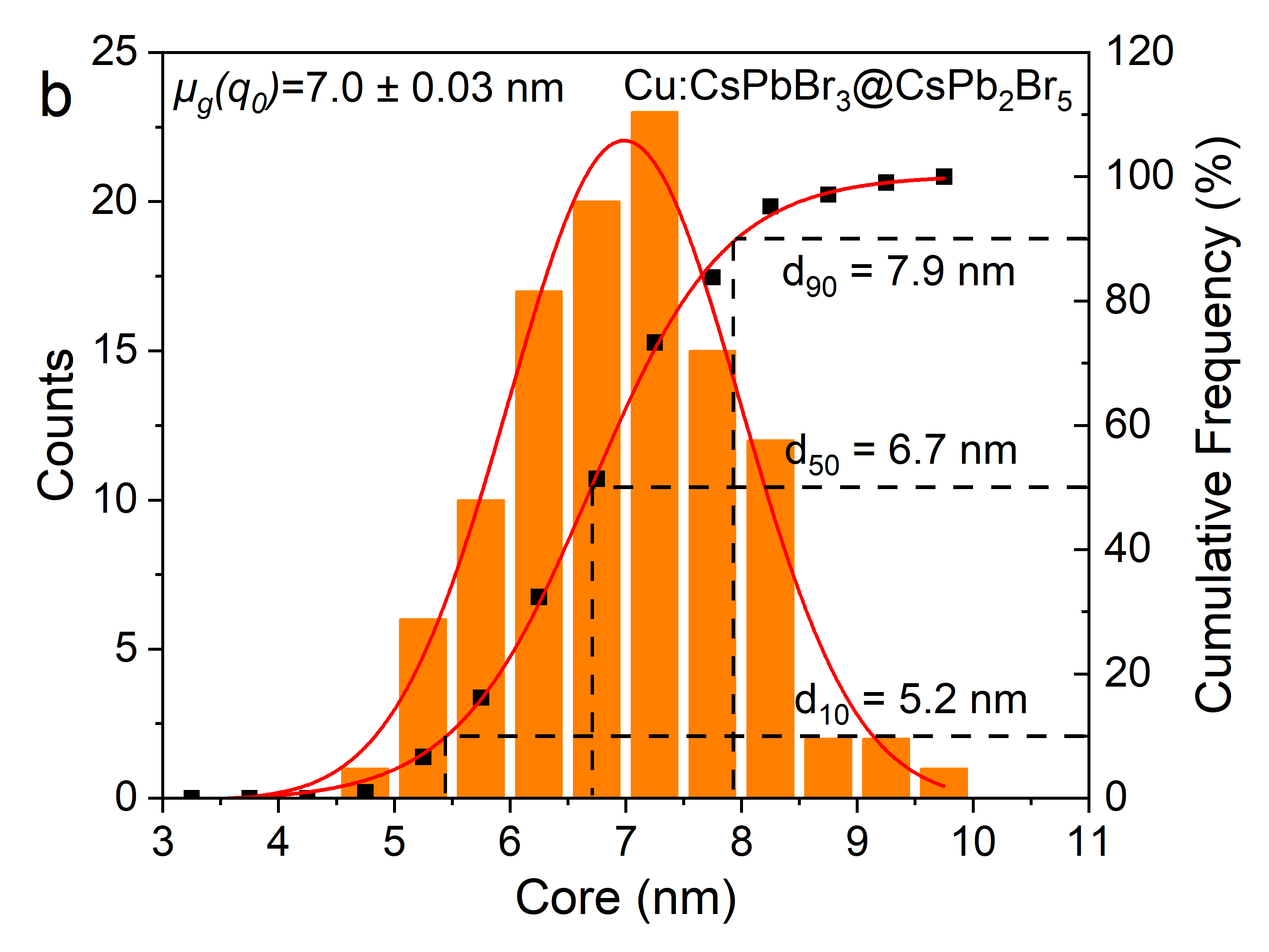


**Figure S1.** Size distribution ofCu:CsPbBr_3_@CsPb_2_Br_5_ (a) shell, (b) core.


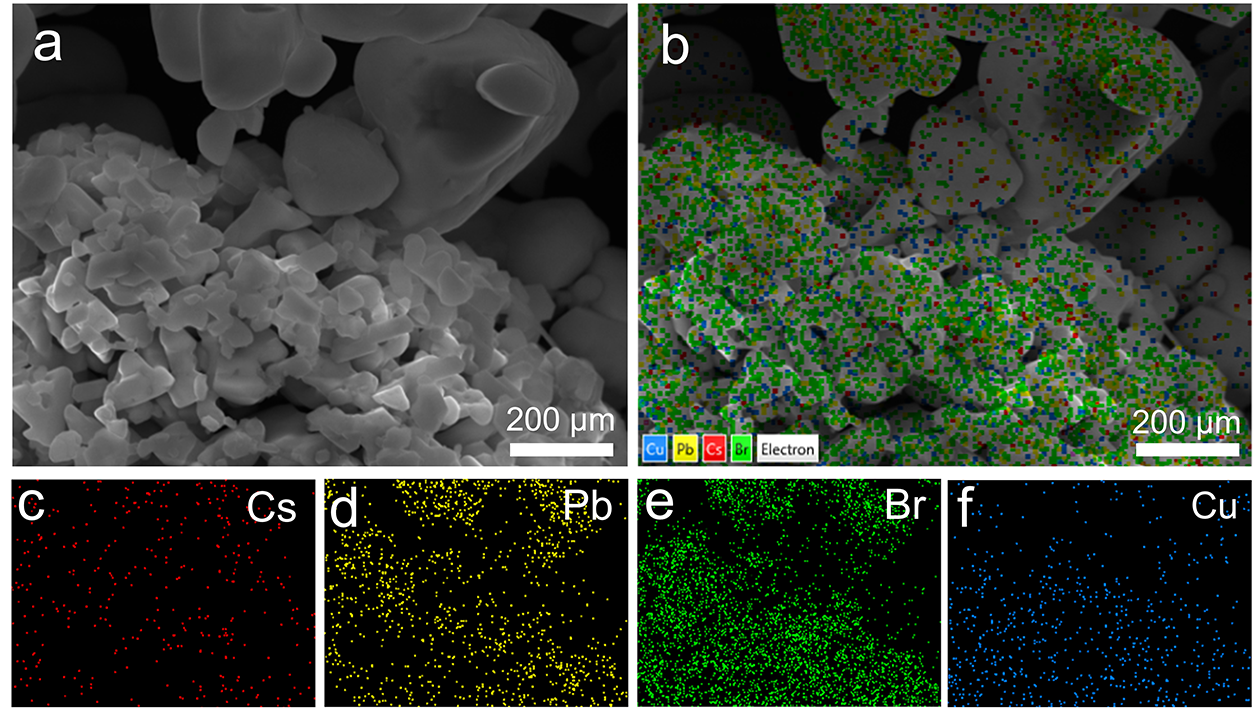


**Figure S2.** (a, b) SEM images of Cu:CsPbBr_3_@CsPb_2_Br_5_. (c-f) Element mapping of Cu:CsPbBr_3_@CsPb_2_Br_5_.


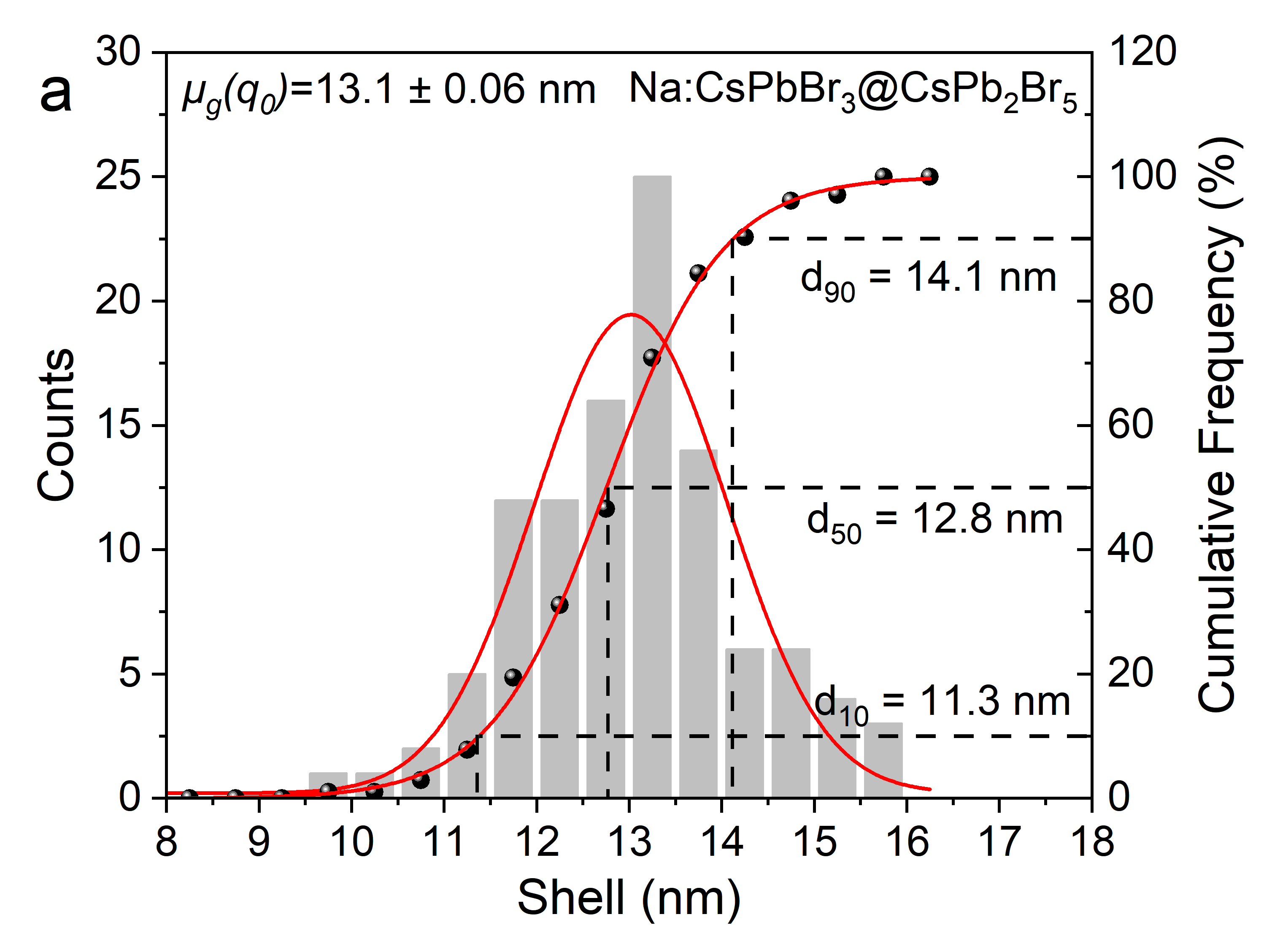

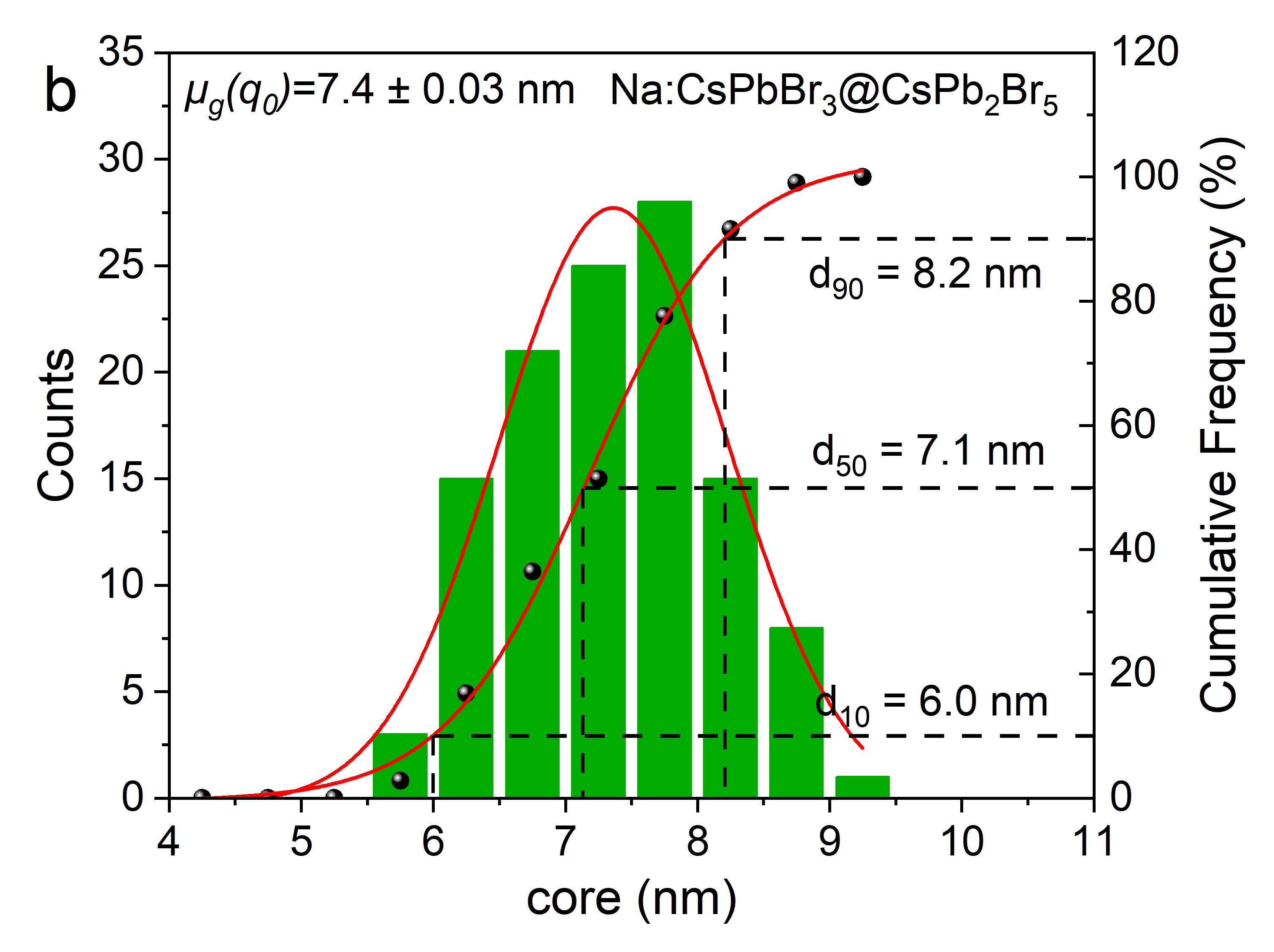


**Figure S3.** Size distribution of Na:CsPbBr_3_@CsPb_2_Br_5_ (a) shell and (b) core.


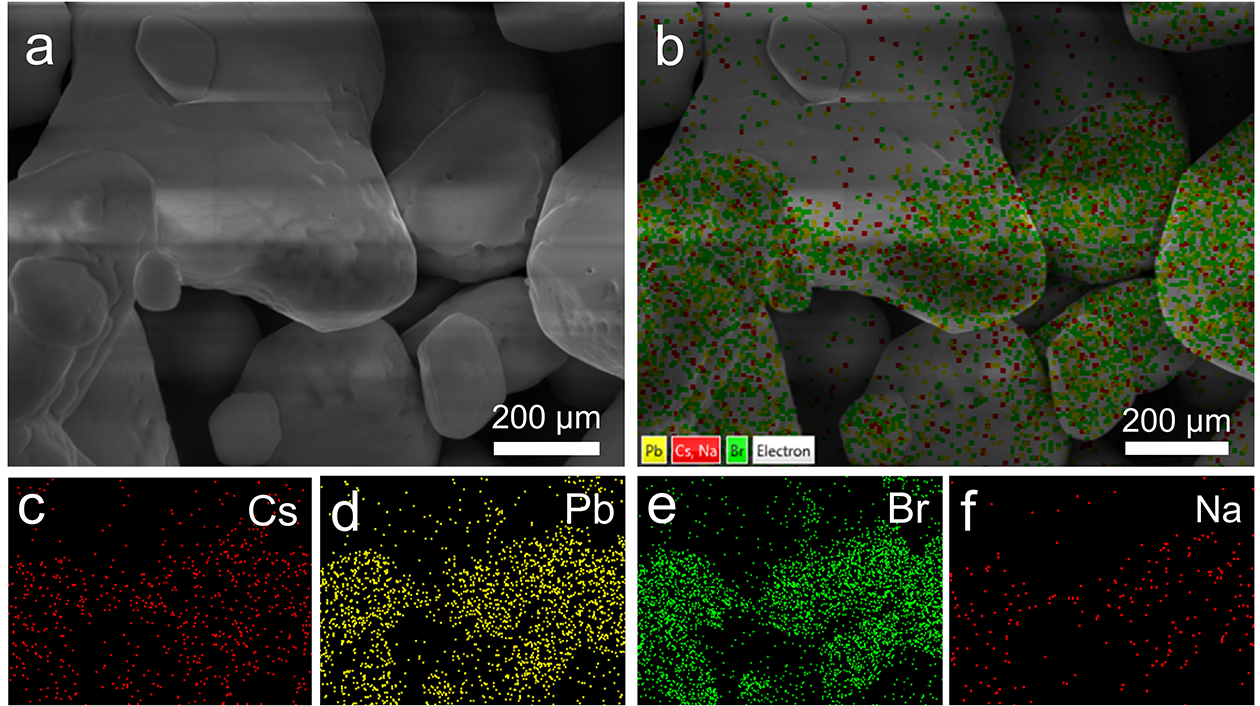


**Figure S4.** (a, b) SEM images of Na:CsPbBr_3_@CsPb_2_Br_5_. (c-f) Element mapping of Na:CsPbBr_3_@CsPb_2_Br_5_.


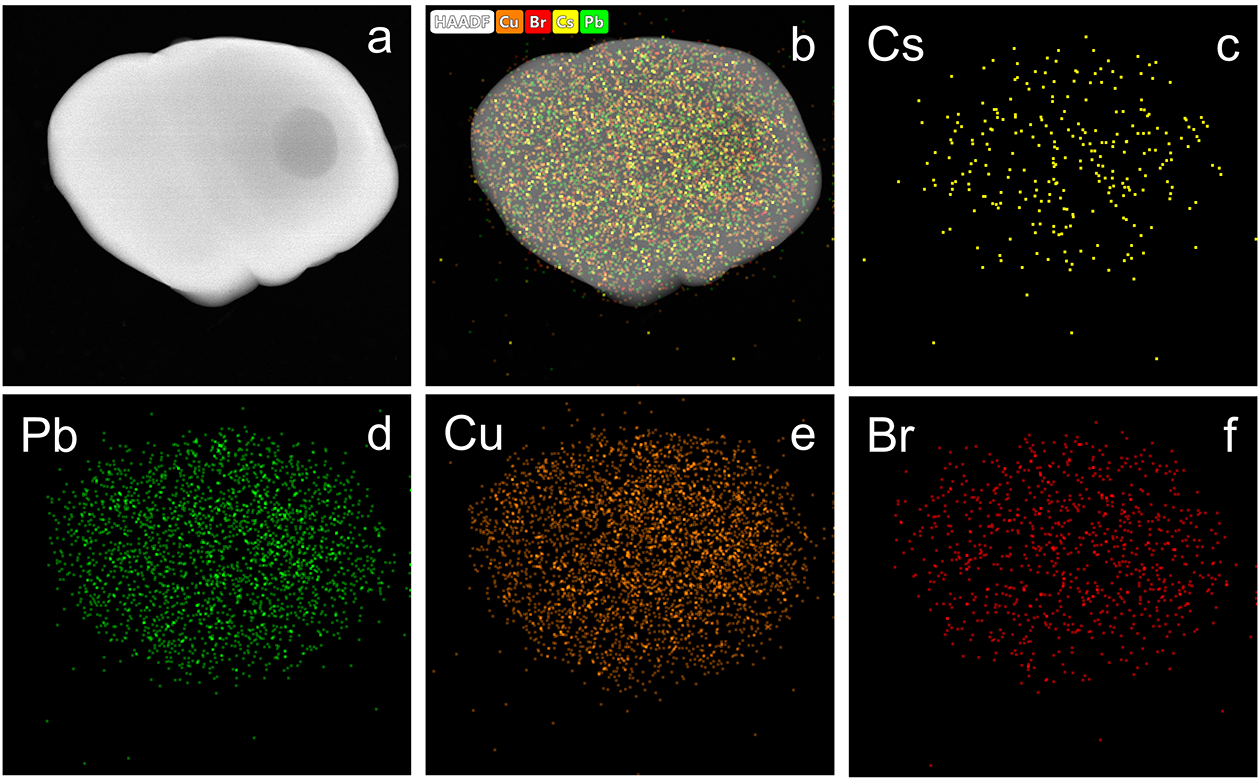


**Figure S5.** (a, b) HAADF-STEM image of Cu:CsPbBr₃@CsPb₂Br₅ nanostructure. (c-f) Corresponding EDS elemental mapping images of Cs (c), Pb (d), Cu (e), and Br (f).


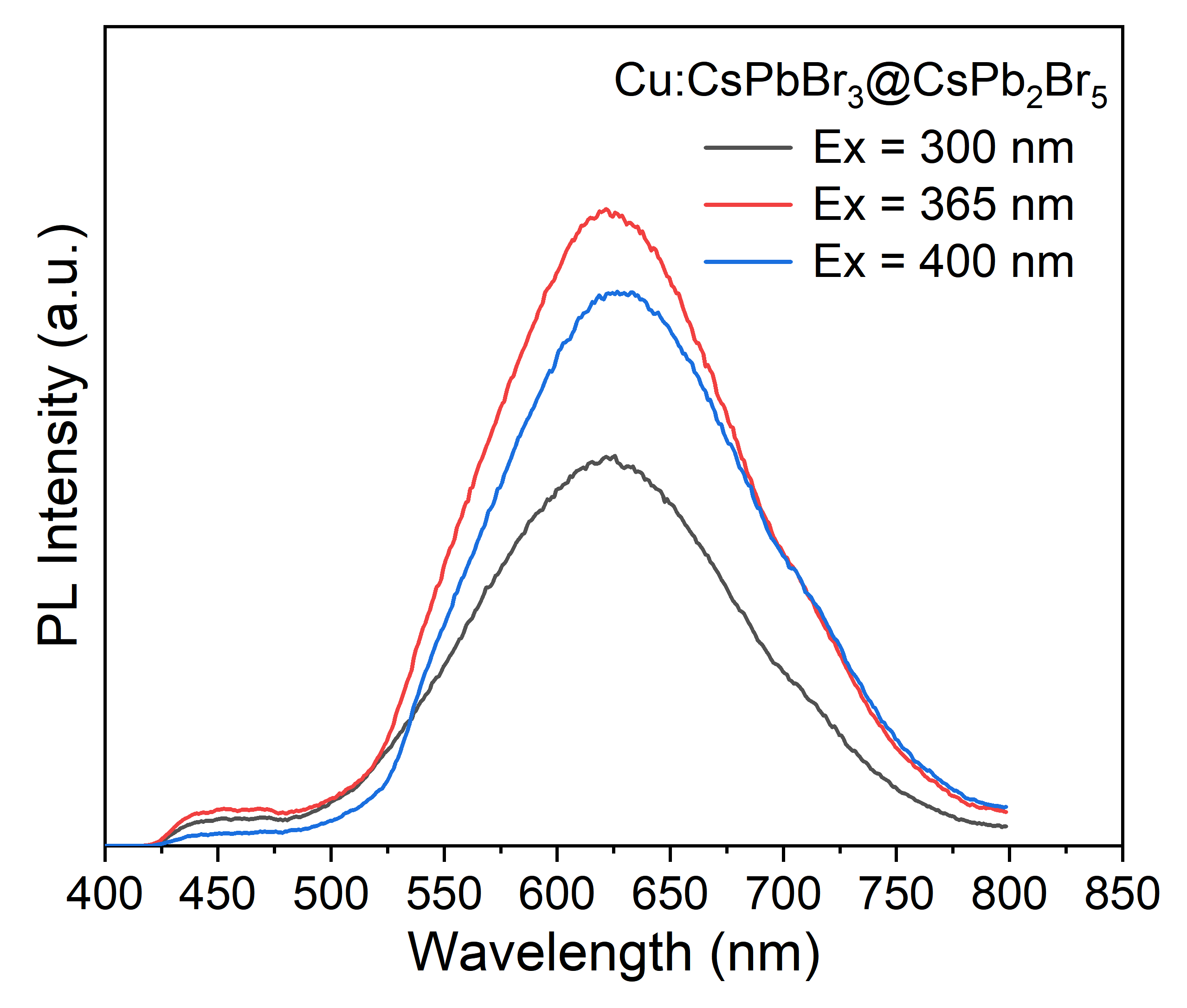


**Figure S6.** PL spectra of Cu:CsPbBr_3_@CsPb_2_Br_5_ samples under different excitation wavelengths.


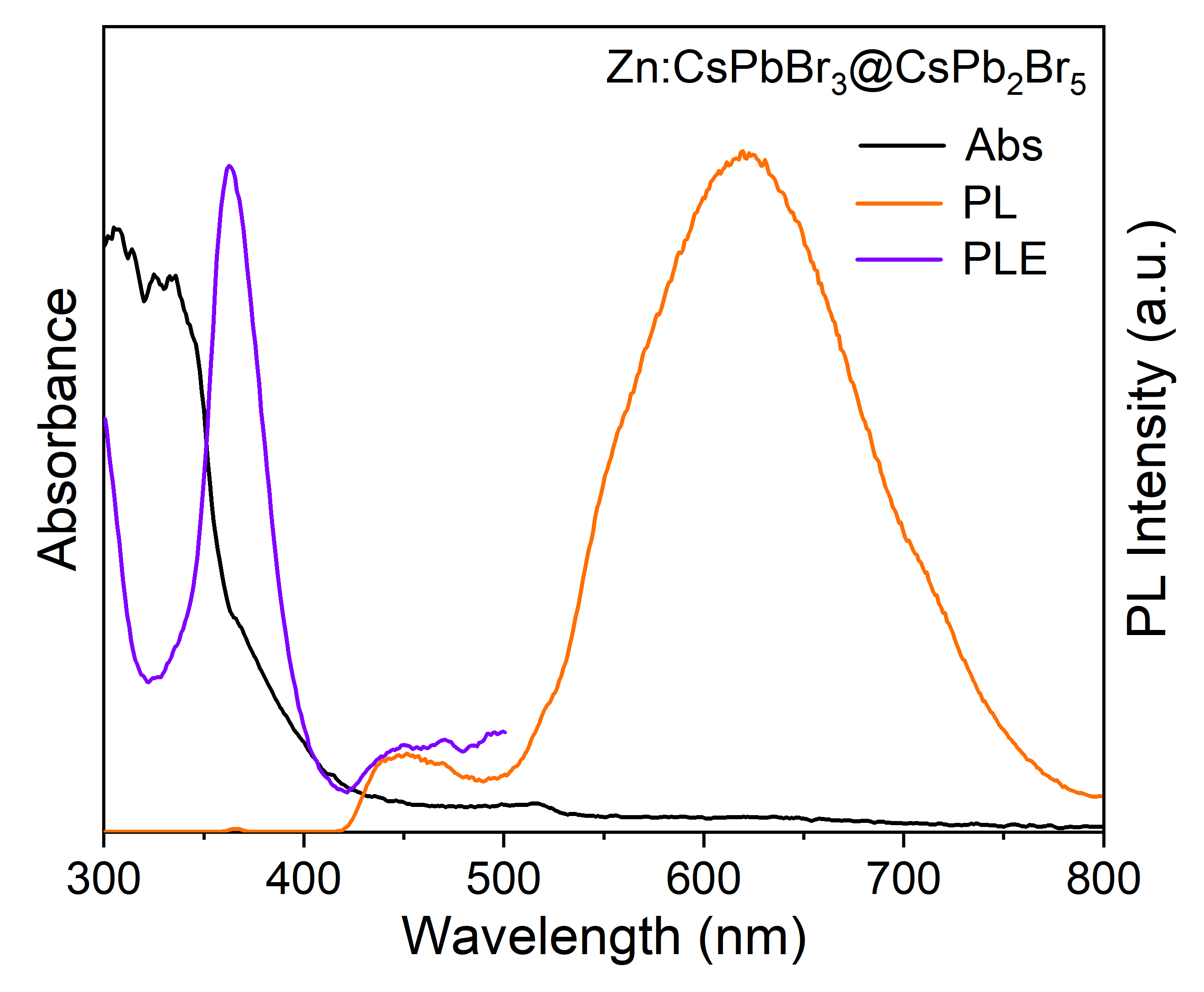


**Figure S7.** DRS, PL and PLE spectra of Zn:CsPbBr_3_@CsPb_2_Br_5_.


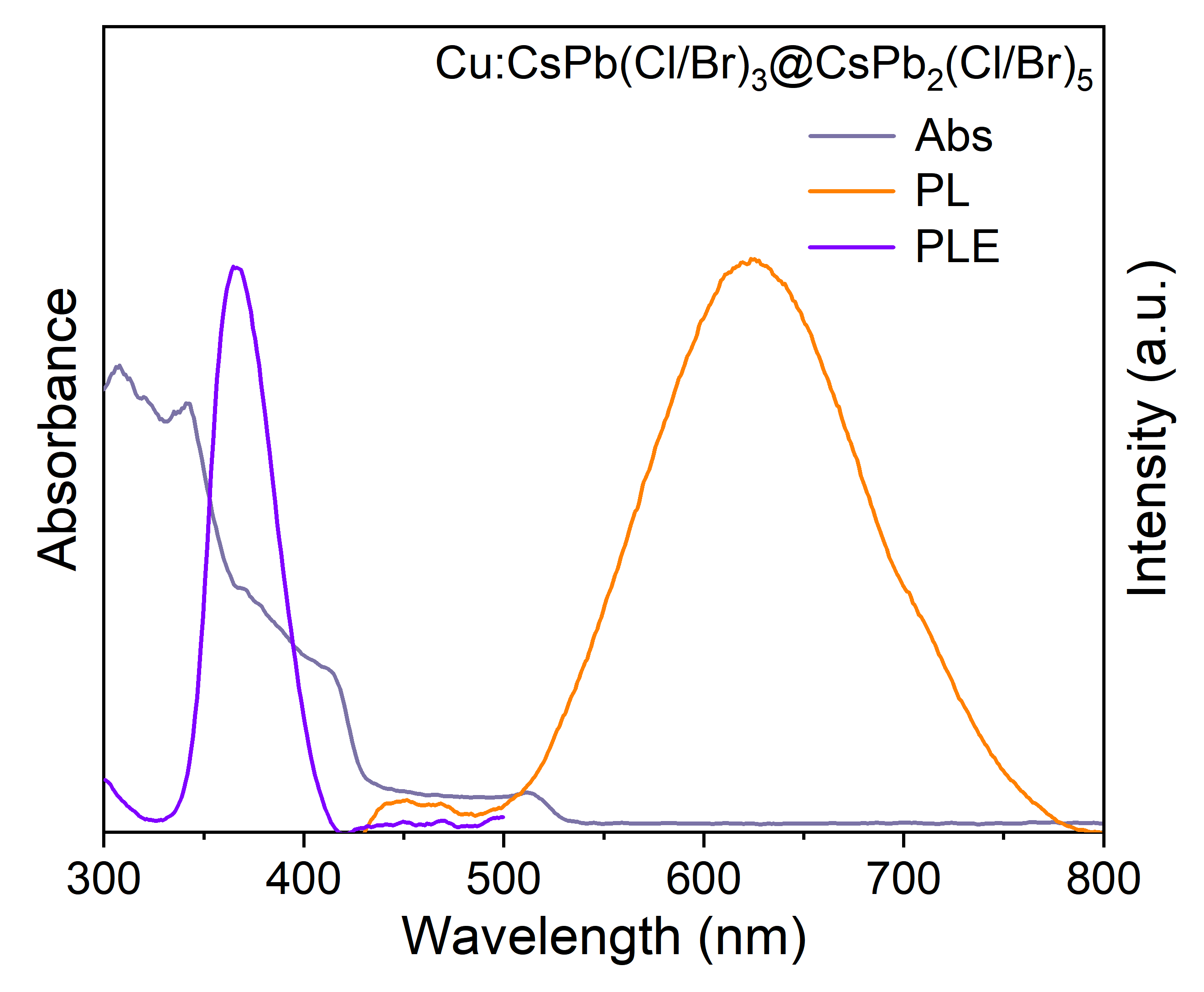


**Figure S8.** DRS, PL and PLE spectra of Cu:CsPb(Cl/Br)_3_@CsPb_2_(Cl/Br)_5_.


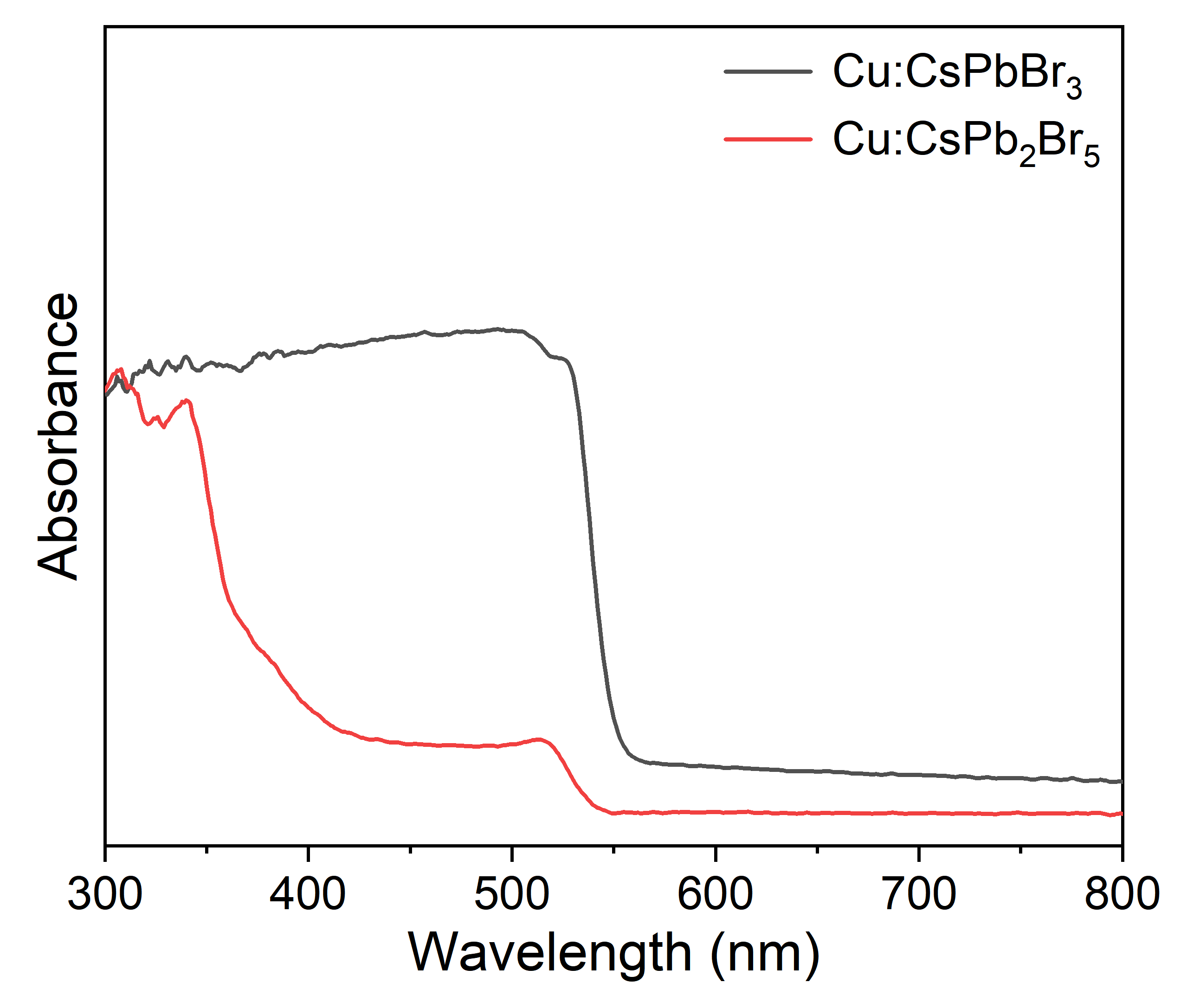


**Figure S9.** DRS spectra of Cu:CsPbBr_3_and Cu:CsPbBr_3_@CsPb_2_Br_5_.


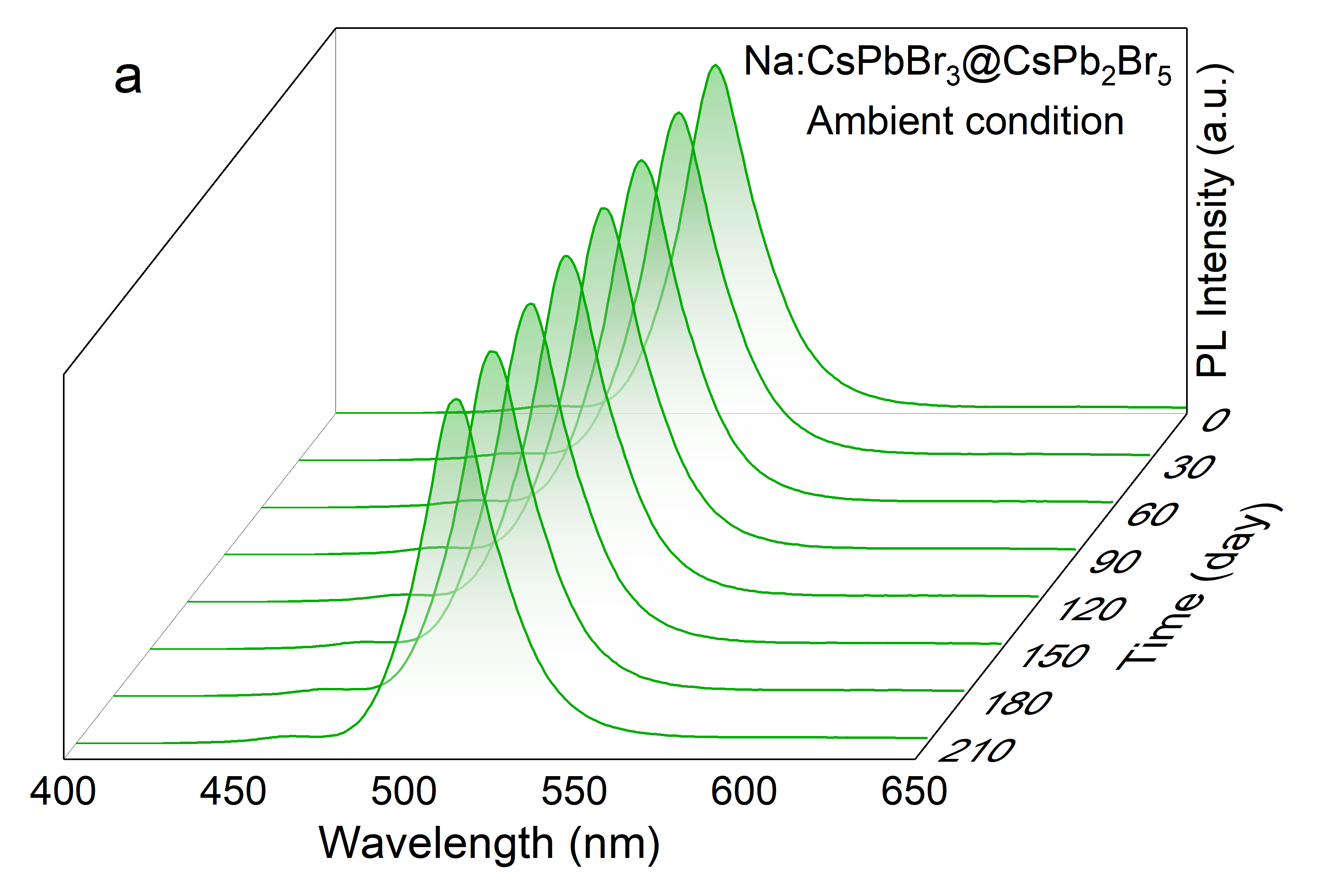

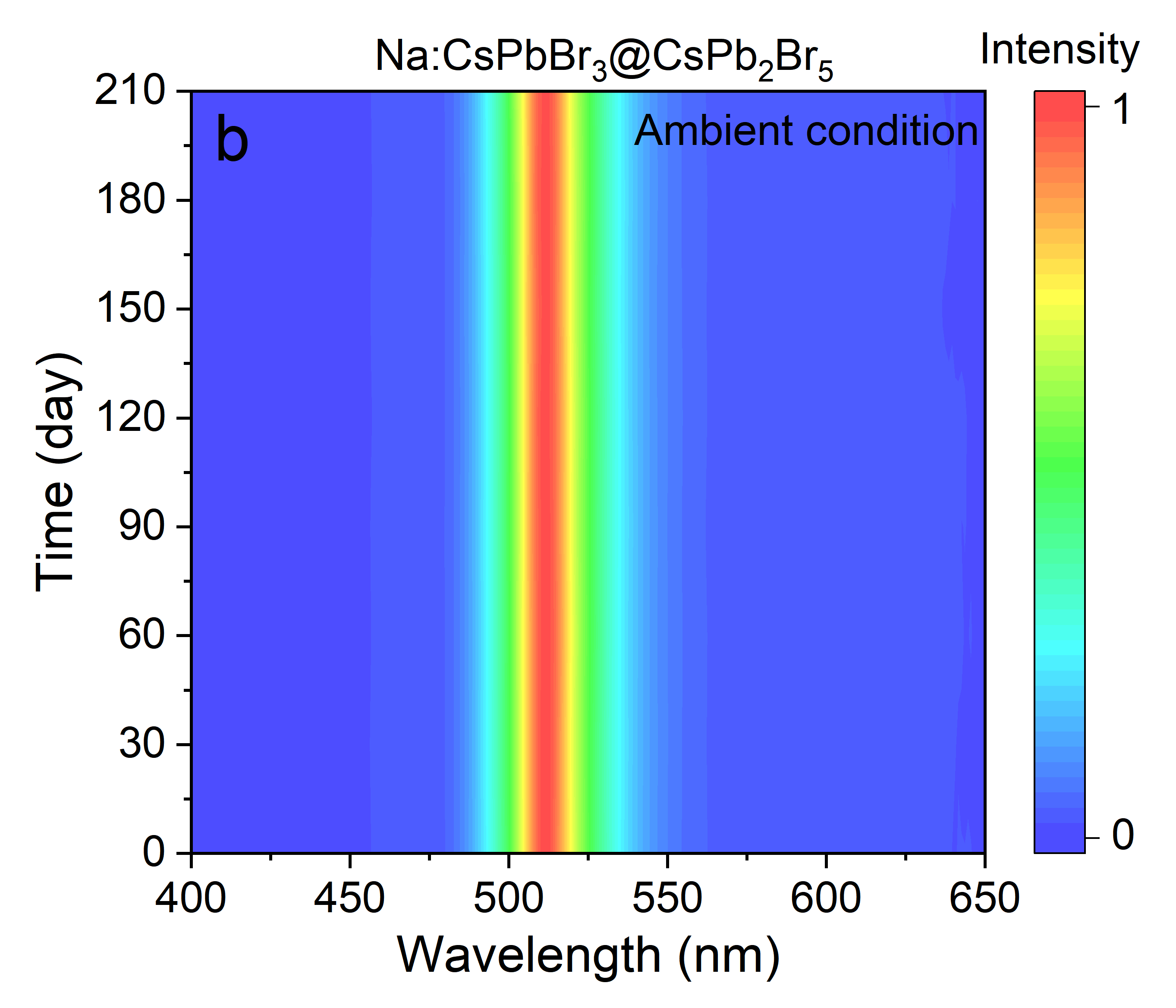


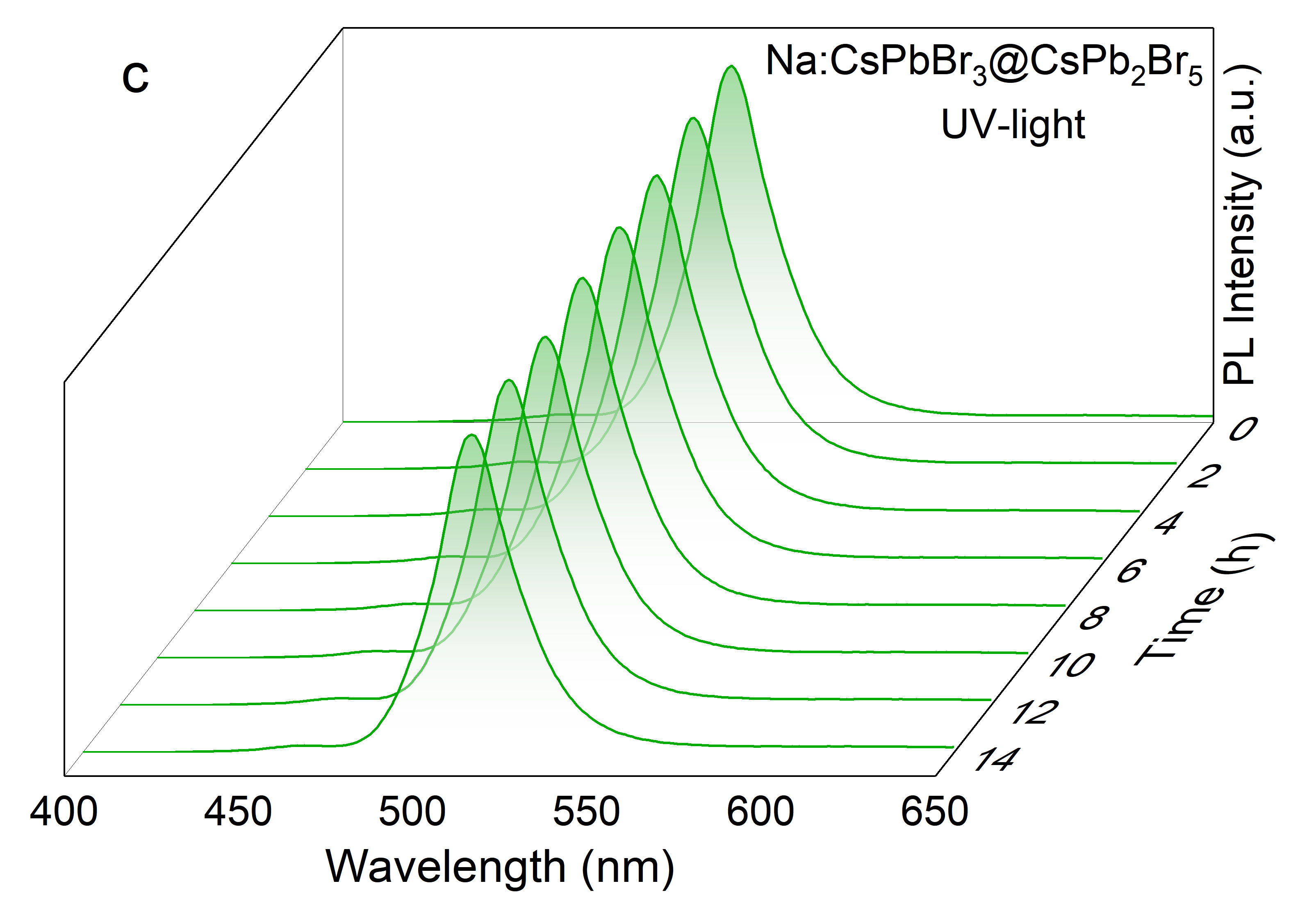

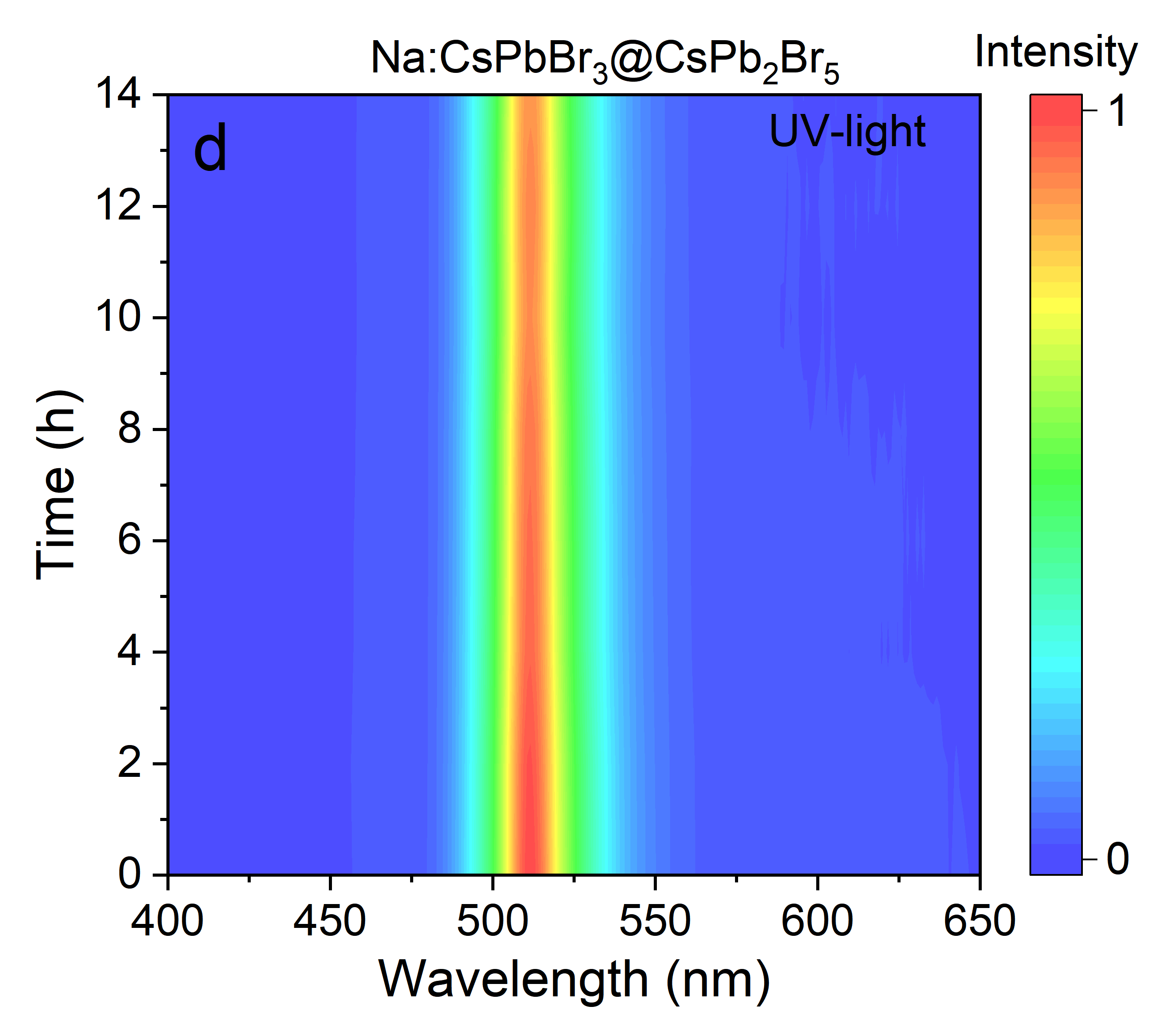

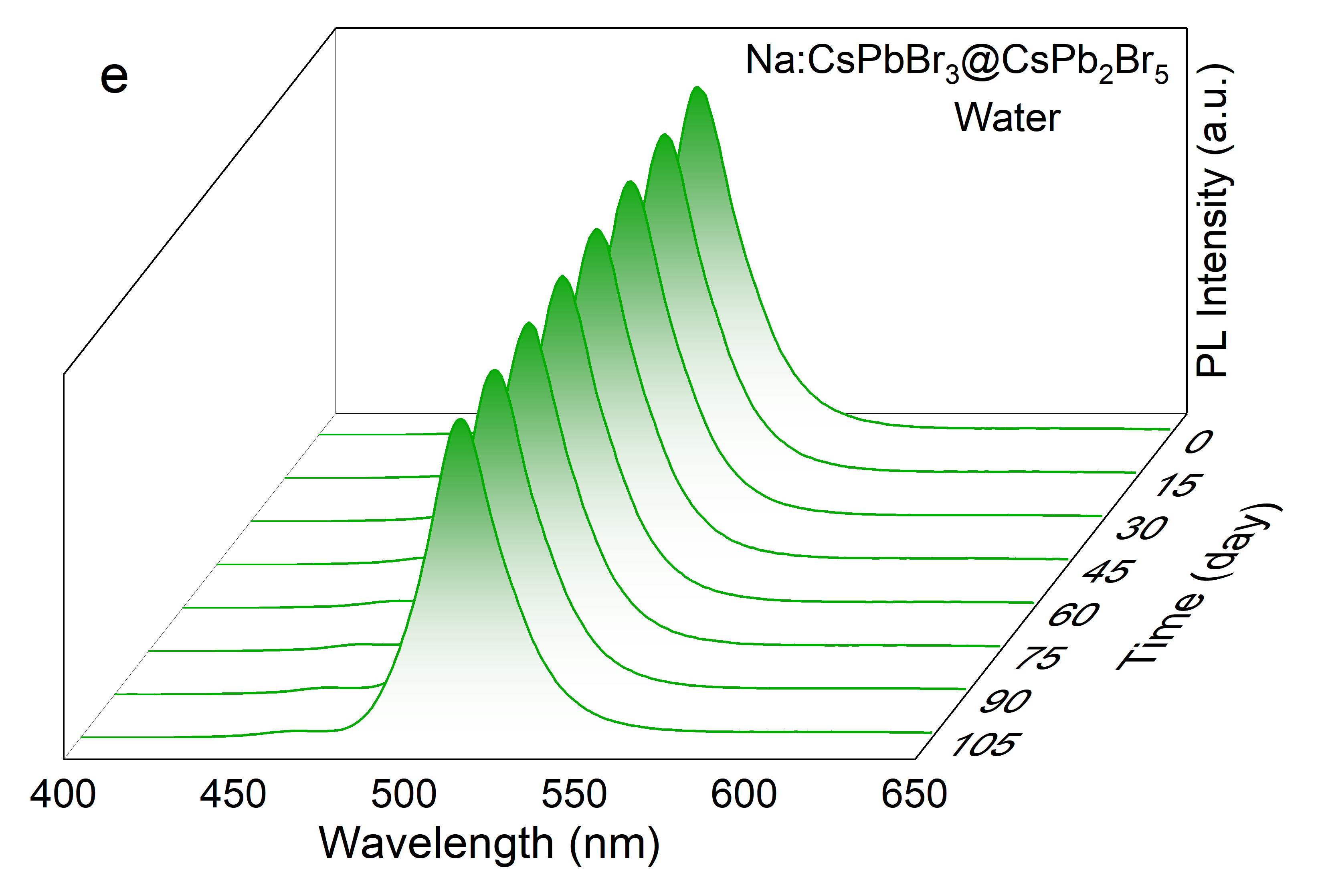

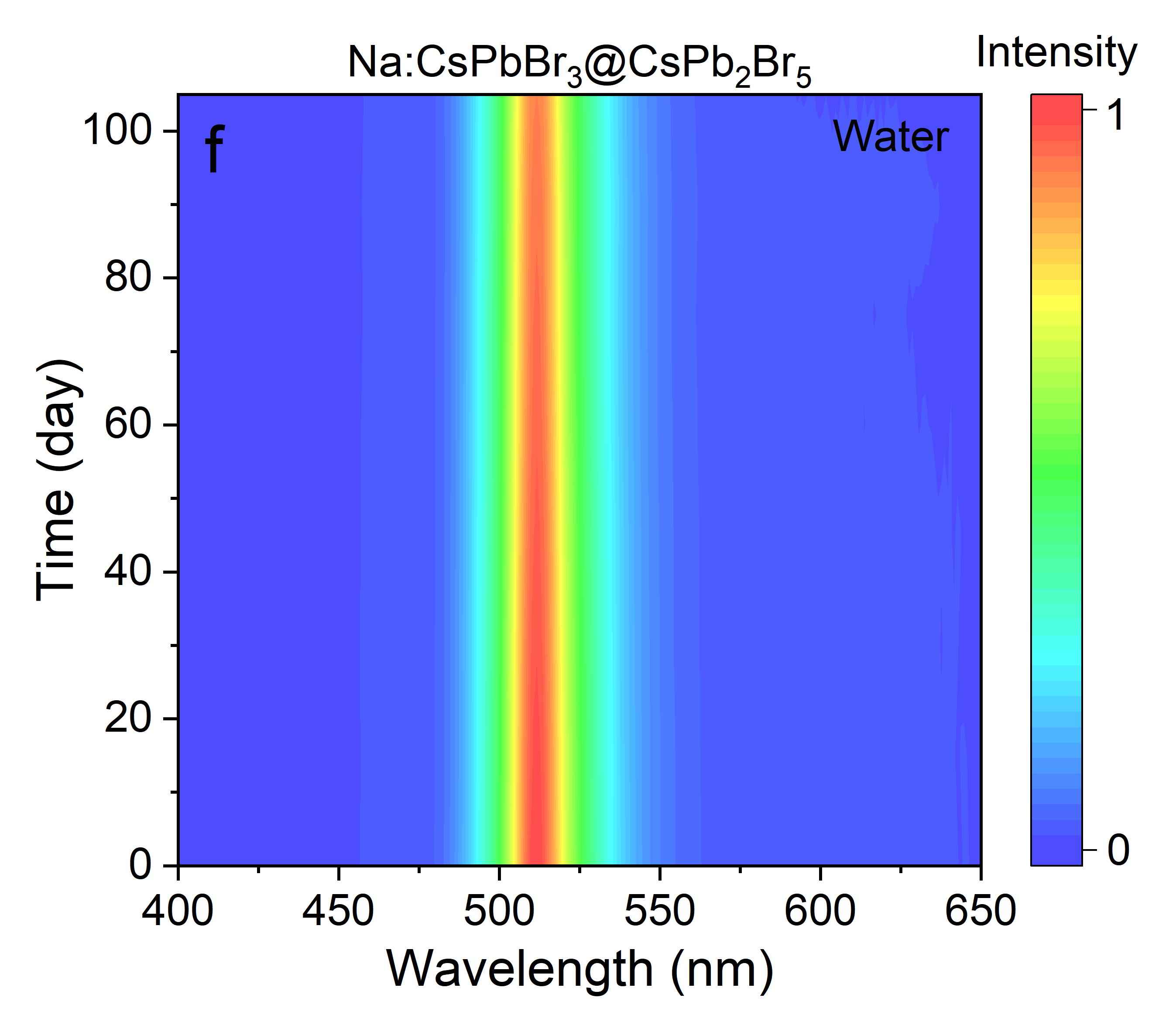


**Figure S 10.** PL stability test of Na:CsPbBr_3_@CsPb_2_Br_5_. (a, b) Ambient condition. (c, d) UV-light irradiation. (e, f) Water treatment.
